# Reaching everyone in general practice? Feasibility of an integrated domestic violence training and support intervention in primary care

**DOI:** 10.1186/s12875-020-01297-5

**Published:** 2021-01-12

**Authors:** Eszter Szilassy, Jessica Roy, Emma Williamson, Katherine Pitt, Mei-See Man, Gene Feder

**Affiliations:** 1 Centre for Academic Primary Care, Bristol Medical School, University of Bristol, Canynge Hall, 39 Whatley Road, Bristol, BS8 2PS UK; 2 School for Policy Studies, University of Bristol, Bristol, UK; 3 Norwich Medical School, University of East Anglia, Norwich, UK

**Keywords:** Domestic violence and abuse, Male and female victims, Male and female perpetrators, Children and young people, Training, Intervention, General practice, Primary care, Feasibility study

## Abstract

**Background:**

Primary care needs to respond effectively to patients experiencing or perpetrating domestic violence and abuse (DVA) and their children, but there is uncertainty about the value of integrated programmes. The aim of the study was to develop and test the feasibility of an integrated primary care system-level training and support intervention, called IRIS+ (Enhanced Identification and Referral to Improve Safety), for all patients affected by DVA. IRIS+ was an adaptation of the original IRIS (Identification and Referral to Improve Safety) model designed to reach female survivors of DVA.

**Methods:**

Observation of training; pre/post intervention questionnaires with clinicians and patients; data extracted from medical records and DVA agency; semi-structured interviews with clinicians, service providers and referred adults and children. Data collection took place between May 2017 and April 2018. Mixed method analysis was undertaken to triangulate data from various sources to assess the feasibility and acceptability of the intervention.

**Results:**

Clinicians and service providers believed that the IRIS+ intervention had filled a service gap and was a valuable resource in identifying and referring women, men and children affected by DVA. Despite increased levels of preparedness reported by clinicians after training in managing the complexity of DVA in their practice, the intervention proved to be insufficient to catalyse identification and specialist referral of men and direct identification and referral (without their non-abusive parents) of children and young people. The study also revealed that reports provided to general practice by other agencies are important sources of information about adult and children patients affected by DVA. However, in the absence of guidance about how to use this information in patient care, there are uncertainties and variation in practice.

**Conclusions:**

The study demonstrates that the IRIS+ intervention is not feasible in the form and timeframe we evaluated. Further adaptation is required to achieve identification and referral of men and children in primary care: an enhanced focus on engagement with men, *direct* engagement with children, and improved guidance and training on responding to reports of DVA received from other agencies.

**Supplementary Information:**

The online version contains supplementary material available at 10.1186/s12875-020-01297-5.

## Background

Domestic violence and abuse (DVA) is a major public health and clinical challenge to the NHS (National Health Service) and health services worldwide [1–3]. It is associated with a wide range of physical and mental health conditions in victims, perpetrators, and their children [4–9] resulting in increased use of health services by all patient groups affected by abuse [1] and vast social and economic costs [10, 11].

The prevalence of DVA among women attending general practice, as in other clinical services, is higher than in the general population [12, 13]. A cross-sectional survey in UK general practice waiting rooms found that 17% of women had experienced physical violence in the past year from a partner or former partner, and 41% had experienced it in their lifetime [14]. A UK survey of male general practice patients reported a one in four lifetime prevalence of DVA experience and a one in six lifetime prevalence of perpetration, although only a small minority experienced coercive control and only one in forty have experienced such violence as victims only [15]. Nonetheless, affected male patients were between two to three times more likely to have current symptoms of depression and anxiety [16].

The NHS and health services internationally have not responded adequately to the needs of these patients [17]. There are international [18] and national UK [1] guidelines in place on the healthcare of women experiencing abuse and a growing recognition of the impact of DVA on their health [17, 19]. IRIS (Identification and Referral to Improve Safety), a training and support programme in general practice, improves the identification of female patients and their referral to specialist agencies [20]. The IRIS trial found a three-fold difference between the intervention and control sites in the identification of female patients experiencing DVA and a six-fold difference in referrals to specialist support agencies. IRIS has been commissioned across more than forty areas in the UK.

The benefits of IRIS to individual female survivors [21], as well as its cost-effectiveness [22, 23] are well established. We know from previous research that women experiencing DVA benefit from being (i) identified by general practice professionals; (ii) referred to DVA specialist agencies; (iii) being contacted by an advocate shortly after referral; (iv) and being offered ongoing support during subsequent GP (general practitioner) consultations to help maintain changes they make as a result of referral.

Despite the success of IRIS in the UK and the growing spread of DVA training programmes for health care professionals across countries in the global North [24], there is a considerable scope both internationally and in the UK to enhance clinicians’ skills and ability to respond appropriately to affected men and children. Clinicians often do not recognise men as victims [25] or perpetrators and there is little research evidence on effective DVA interventions for men in the healthcare setting [26, 27]. There is also uncertainty about the healthcare response to DVA when children are affected [28–32], and about the effectiveness and cost-effectiveness of interventions to improve healthcare responses to children experiencing DVA [33].

A systematic review found that training interventions with system support benefit victims [34] and we know that general practice clinicians want integrated DVA training [35] alongside a programme that includes advocacy for men as perpetrators and victims [36]. The evidence reviews in the UK National Institute for Health and Care Excellence DVA guidelines [1] identified gaps in the evidence for an integrated healthcare response and for interventions for perpetrators.

The IRIS+ (Enhanced Identification and Referral to Improve Safety) primary care intervention enlarged the original IRIS model beyond women survivors of DVA to also address the needs of men (survivors and perpetrators) and their children. The aim of this study was to test the feasibility and acceptability of IRIS+, consistent with the MRC guidance on evaluation of complex interventions [37, 38], using mixed method evaluation: capturing DVA identification and referral data and an assessment of clinician and patient engagement with and experience of the intervention.

Here we report key findings of the feasibility study and identify areas for intervention improvement, as well as future research directions. Given the well established evidence on the feasibility and effectiveness of IRIS to female survivors [20–22], this paper primarily focuses on the implications of the IRIS+ feasibility findings to men and children. Detailed findings of individual evidence streams [25, Huntley et al., Help seeking by male victims of domestic violence and abuse: an integrated mixed method analysis of systematic review evidence, accepted] specific, areas of investigation [Roy et al., ‘It felt like there was always someone there for us’: supporting children affected by domestic violence and abuse in general practice, under review] and findings of an associated sub-study [39] are reported elsewhere.

## Methods

### Intervention development

The multidisciplinary research team integrated diverse evidence sources into the development of the IRIS+ training and support intervention. These included (i) a systematic review and thematic synthesis of qualitative studies [25], and (ii) an integrated mixed method analysis of systematic review evidence of help-seeking by male victims of DVA [Huntley et al., Help seeking by male victims of domestic violence and abuse: an integrated mixed method analysis of systematic review evidence, accepted]; (iii) a mapping study of UK services for male victims; (iv) a two-stage consensus process with a multi-professional stakeholder group including experts of DVA and health; (v) and a qualitative sub-study with general practice clinicians and police staff about information sharing [39]. The design of the intervention was also informed by previous research evidence [20, 36, 40], particularly our study of prevalence of DVA in male general practice populations [16], a systematic review on interventions aimed to professionals working with children exposed to DVA [41], as well as our systematic reviews of DVA and mental health [42, 43].

Building on the IRIS model, a key component of the IRIS+ intervention was the development of specialist DVA support worker roles. The IRIS+ advocate educator (AE) and IRIS+ children and young persons’ worker (CYPW) roles were informed by review of current evidence [25, 33, 44, 45, Huntley et al., Help seeking by male victims of domestic violence and abuse: an integrated mixed method analysis of systematic review evidence, accepted] and following extensive consultations with stakeholders.

**Fig. 1:**
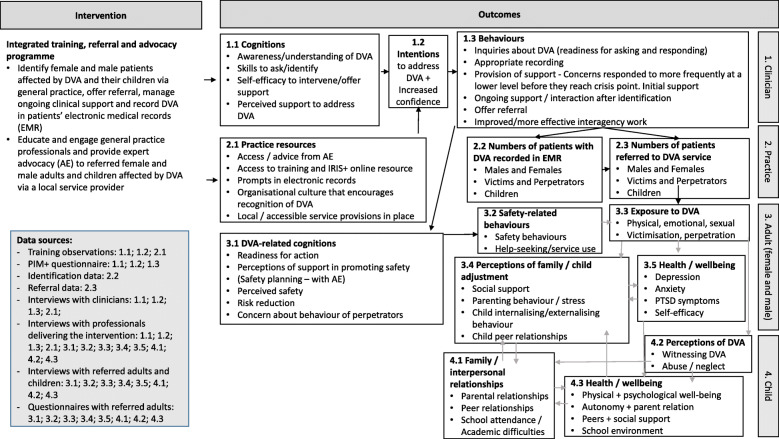
IRIS+ logic model

A core part of the process was the development of a logic model to be used to describe the key elements of the intervention and the activities employed to addresss these. The model also outlined how the intervention mapped onto measurable core outcome domains and key interactions between them. It described expected proximal or long term outcomes corresponding to these domains for different participant groups directly or indirectly involved with or affected by the intervention. The logic model (Fig. 1) highlighted potential data sources along the causal pathway towards outcomes for survivors of DVA and their families which we used to evaluate the IRIS+ model.

**Fig. 2:**
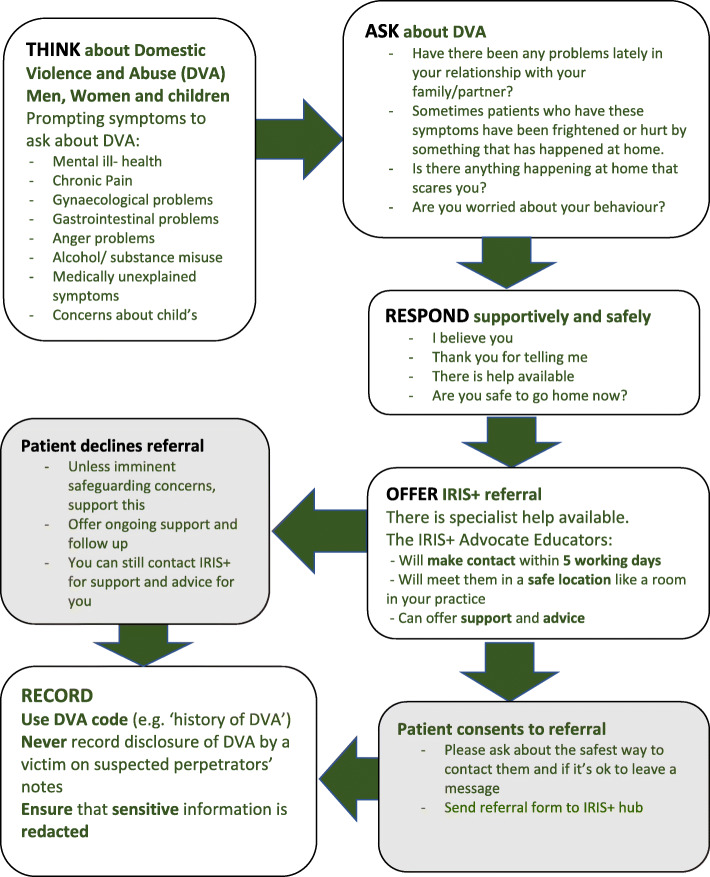
Summary of the IRIS+ care and referral pathway for clinicians

Work also included the development of an online resource freely accessible by clinicians receiving the IRIS+ training. The resource was intended to supplement and consolidate face-to-face learning by providing easy access to key components of the training and practical information. A system of electronic prompts triggered by codes for health conditions associated with DVA has been also developed, but implementation failed due to system-wide infrastructure problems that remained unresolved during the study period. Additional work consisted the development of IRIS+ publicity materials. These included patient- and clinician-facing materials (patient waiting room poster, patient card, care and referral pathway clinical summary, clinician mouse mat with useful information, points of advice and referral contact details) (see Fig. 2).

### IRIS+ intervention

The IRIS+ training and support intervention was designed to engage general practices by extending the IRIS model and including elements of training around the needs of affected men [36] and children [46]. It aimed to increase the safety and wellbeing of families affected by DVA by improving how general practice professionals respond to female and male adult patients who experience or perpetrate DVA and to their children who may have been living with DVA.

The two times two-hour face-to-face interactive training intervention was designed to improve the identification and management of DVA in general practice by (i) consolidating and improving general practice clinicians’ knowledge of DVA and health; (ii) enabling them to identify, respond to and support female and male patients affected by DVA and their children; (iii) developing a good understanding and knowledge of referral routes and enabling them to offer referral for all family members affected by DVA; (iv) preparing clinicians to safely and accurately record DVA in patients’ electronic medical records (EMR); and (v) equipping them to manage ongoing relationships with affected patients including members of the same family (Fig. 2).

The training intervention included an additional brief (up to half an hour) reminder and question and answer session during a clinical practice meeting four to six months after the delivery of the second training session and a one-hour information session for reception and administrative staff.

The AE and the CYPW were based in the IRIS+ hub in a voluntary sector DVA agency, delivering the training, receiving referrals from clinicians, and providing expert advocacy to referred female and male adults and children affected by DVA. For male perpetrators, following an initial risk assessment, the options included sign-posting to other support services and participation in a perpetrator group programme.

### Intervention delivery

IRIS+ was implemented in an urban area in England across four general practices that had not previously received IRIS or other GP practice team-based DVA interventions. The two training sessions were delivered between two to four weeks apart during May–July 2017 to whole practice clinical teams. These, as well as the reminder sessions, were jointly delivered by a local GP with specific expertise on DVA, an AE with clinician training experience linked to the support agency, and a local social worker representing children social care.

### Evaluation of feasibility

We used a range of methods to assess the feasibility and acceptability of the IRIS+ intervention. These included (i) training observations; (ii) measuring change in clinicians’ preparedness through a pre/post questionnaire (PIM+); (iii) DVA identification data extracted pre-and post-intervention from the EMR of the participating practices; (iv) IRIS+ referral and service support contact data extracted from the third sector partner agency; (v) semi-structured interviews with participating clinicians; (vi) semi-structured interviews with professionals delivering the intervention; (vii) semi-structured interviews with referred adults and children; and (viii) questionnaires with referred adults. Data collection took place between May 2017 and April 2018.

(i) All the training sessions were observed by a researcher guided by an observation framework. The observations documented the context and dynamics of training sessions, variations in training delivery, and participants’ engagement with and reflections on the content and teaching methods. The observations helped us explore factors which may have facilitated or limited change in individual or collective practices.
(ii) The PIM+ questionnaire (see additional files 1 and 2) was developed from the PIM (PROVIDE Intervention Measure) questionnaire [36] and was adapted to include questions relevant to IRIS+ about clinicians’ perceived preparedness to perform various key tasks relevant to DVA (ask patient groups about DVA, identify signs and symptoms of abuse, validate disclosures, offer referrals, safely record DVA, provide ongoing support). The survey also collected general demographic and workload-related information about the training participants, and information on the number of DVA identifications they made in different patient groups in the previous six months. The questionnaire used in our study was not a fully validated measure, although it had reasonable test-retest reliability. Clinicians undertaking the training in the four pilot practices were asked to complete the online survey before the first training and again after nine months. Twenty-five of thirty invited clinicians completed the survey, with 18 completing it at both time points.
(iii) Data were extracted from the EMR for a period of ten months after the delivery of the first IRIS+ training intervention to measure clinical DVA identifications during the study period. We searched for specific codes relating to DVA victimisation or perpetration. Cases that were identified were individually checked for DVA relevance and for the action taken by the clinician. We also extracted data on gender, age, and number of children.
(iv) The agency hosting the IRIS+ service (IRIS+ hub) collected data on the number of patient referrals from the pilot practices, as well as client contact over the course of the study. Data on the source of referrals, the number, type and duration of client contacts (adults and children) with the AE and CYPW as well as victim/perpetration status, and non-identifiable demographic information on age, gender, ethnicity and number of children were collated and passed onto the research team for analysis.
(v) We conducted semi-structured interviews with clinicians (GPs and nurses) participating in the training intervention at two time points, soon after the IRIS+ training (T1, *n* = 9) and between six and nine months after the second training (T2, *n* = 8). Eight clinicians completed interviews at both time points. The interviews focused on clinicians’ experience of the IRIS+ intervention, their views on the service and what enablers and barriers they experienced during implementation (see additional file 3).
(vi) We conducted semi-structured interviews with IRIS+ professionals (GP trainers, AEs, CYPW) delivering the training and support intervention (*n* = 5). Interviews focused on professionals’ views on clinician and patient engagement with the training and support intervention and how the IRIS+ intervention had been received and implemented in practice (see additional file 4.).
(vii) We conducted semi-structured interviews with referred adult patients soon after their referral/first meeting with the IRIS+ AE (*n* = 12) and upon completion of their support intervention, three to six months later (*n* = 8) (see additional file 5). We also conducted semi-structured interviews with children receiving direct support (*n* = 3) upon support completion. We asked adult interview participants about their experiences of being referred to the IRIS+ hub and their experiences of receiving support. We also asked parents about the health and wellbeing of their children and what impact any direct or indirect support had had upon them. Children were recruited via their non-abusive parent who, in turn, were introduced to the study by the AE. The children’s interviews focused on the children’s experience of receiving support from the CYPW (see additional file 6).
(viii) At the same time as conducting the interviews, we also administered questionnaires with referred adult patients (baseline *n* = 12; follow-up *n* = 8). In total seven adult patients completed both the baseline and follow up interviews and questionnaires. The questionnaires contained a combination of validated and non-validated measures. They elicited socio-demographic details, information on past and current experiences and impact of DVA, physical and mental health, quality of life, use of health services, and their children’s health and wellbeing (see additional file 7).

All professional and patient interviews were audio-recorded, transcribed verbatim, loaded into qualitative data analysis software (NVivo v.11) and analysed thematically [47] using a coding frame incorporating concepts that emerged from the data. Data in (ii)-(iv) were analysed descriptively in Stata (v15). Due to small sample size, the study did not aim to draw inferences from quantitative data.

For the mixed method analysis, we used a convergent design where we first independently analysed quantitative and qualitative data and then used triangulation to refine our coding frame and map qualitative dimensions of feasibility and acceptability to data from questionnaires. We also checked our process data against our logic model to track intervention flow and determine whether there was evidence that steps in the logic model were being reached in the study. We refer to each step (see corresponding numbers in Fig. 1.) in the ‘Results’ section and describe how these relate to data sources and contribute evidence for or against the feasibility of the intervention.

The convergent approach helped to identify instances of inconsistency, assess the suitability of our service-level data capture protocols, and the validity of questionnaire data at the level of individual research participant. Cross-mapping analytical frameworks around key themes and connecting qualitative and quantitative methods of data interpretation facilitated the emergence of new insights beyond those identified through separate analysis of various data components [48].

We engaged closely with three service user expert groups (female survivor, male victim, male perpetrator) from the development of the original proposal, through protocol development, writing of participant recruitment materials, development of the intervention, conduct of the study, and interpretation and dissemination of the findings.

## Results

### Reaching clinicians

#### Engagement with and acceptability of the IRIS+ intervention

The delivery of the IRIS+ intervention proved acceptable to general practice teams and service providers (Fig. 1. Box 2.1). Clinicians gave positive feedback on training delivery and content. Participants found the information about the impact of DVA on patients’ health valuable and the focus on all family members (not just women) beneficial. As noted by a GP:

*It was useful to know that there is support available for both victims and perpetrators. Having that more rounded view of trying to resolve or help people in domestic abuse situations …* (Clinician2T1).

The service provided by the AE was praised by clinicians, who stated that a named support worker was the key to the service’s acceptability and feasibility*.*

*That’s one of the advantages of having clapped eyes on them and spoken to them. It’s one of the benefits of the training, really, that you have a face that you can put a name to.* (Clinician3T1)

All clinicians reported that the IRIS+ hub had filled a service gap for men and children, had been well organised and easily accessible, and the intervention relevant, safe and acceptable to patients.

*We’ve had much better access. It’s been quick* – noted a GP. (Clinician5T2)

The direct referral route and support for patients and clinicians facilitated DVA recognition:

*You’re probably more likely to identify things because you know there’s a support service there.* (Clinician6T2)

The ability to refer to a programme for male perpetrators facilitated the identification and ongoing support work:

*There is so little available for people who are perpetrating abuse [ … ] actually all you can do is often, as a professional involved, to make suggestions or requirements for the non-abusive parent. IRIS+ is different in that way.* (IRIS+ AE1)

Being able to offer dedicated direct support to children experiencing DVA, without having to negotiate social services referral with families or to judge whether they were above the children’s social care referral threshold, were greately valued by clinicians:

*You feel sometimes that social service referrals are a bit punitive, whereas IRIS+ just feels more supportive for the patients.[…] I think the feeling is that it’s a service that is very useful. […] You realise that things have been under our nose for quite a long time, so I think that’s probably why there are quite a few referrals relatively quickly. (*Clinician6T2*)*

#### Impact on clinical practice

Clinician survey responses completed at both time points indicated that the IRIS+ training had led to consistent perception of improvements in all areas of practice (Fig. 1. Box 1.1; 1.2). Clinicians felt more prepared to ask questions, identify signs and symptoms of DVA and provide appropriate response to disclosures (Table 1.).

**Table 1:** Change in clinicians’ self-reported preparedness to respond to DVA at baseline T1) and follow-up (T2)

PIM+ questionnaire domains	n	T1 mean score	T2 mean score	Median change	95% CI	Signed rank test ***P***-value
**Ask about DVA**						
Female victims	18	2.9	4.3	1.5	[2.0, 1.0]	0.0006
Female perpetrators	18	1.5	3.6	2.0	[2.5, 1.5]	0.0002
Male victims	18	2.1	4.1	2.0	[2.5, 1.5]	0.0002
Male perpetrators	18	1.9	3.6	1.5	[2.5, 1.0]	0.0005
Parents	18	2.1	3.7	1.5	[2.0, 1.0]	0.0002
Children and young people	17	2.3	3.6	1.0	[1.5, 1.0]	0.0004
**Identify signs and symptoms of DVA**						
Female victims	18	3.1	4.4	1.5	[2.0, 1.0]	0.0005
Female perpetrators	18	1.6	3.8	2.0	[3.0, 1.5]	0.0002
Male victims	18	2.2	4.0	2.0	[2.0, 1.5]	0.0002
Male perpetrators	18	2.1	3.9	2.0	[2.5, 1.0]	0.0001
Parents	17	2.4	4.0	1.5	[2.0, 1.0]	0.0003
Children and young people	18	2.6	4.1	1.5	[2.0, 1.0]	0.0002
**Respond to initial disclosure of DVA**						
Female victims	18	2.9	4.4	1.5	[2.0, 1.0]	0.0002
Female perpetrators	18	1.7	4.2	2.5	[3.0, 2.0]	0.0002
Male victims	18	2.5	4.3	2.0	[2.0, 1.5]	0.0002
Male perpetrators	18	1.9	4.2	2.5	[3.0, 1.5]	0.0002
Parents	18	2.4	4.2	1.5	[2.0, 1.0]	0.0002
Children and young people	18	2.7	4.1	1.5	[2.0, 1.0]	0.0004
**Refer**						
Female victims	18	2.9	4.6	1.5	[2.5, 1.0]	0.0005
Female perpetrators	18	1.6	4.2	2.5	[3.5, 2.0]	0.0002
Male victims	18	2.2	4.6	2.5	[3.0, 2.0]	0.0002
Male perpetrators	18	1.9	4.2	2.5	[3.0, 1.5]	0.0003
Parents	18	2.4	4.4	2.0	[2.5, 1.5]	0.0002
Children and young people	18	2.6	4.4	2.0	[2.5, 1.0]	0.0002
**Record information about DVA**						
Female victims	18	2.8	4.2	1.5	[1.5, 1.0]	0.0005
Female perpetrators	18	2.2	4.1	2.0	[2.5, 1.5]	0.0003
Male victims	18	2.6	4.1	1.5	[2.0, 1.0]	0.0003
Male perpetrators	18	2.4	4.0	1.5	[2.5, 1.0]	0.0004
Parents	18	2.6	3.9	1.5	[2.0, 1.0]	0.0006
Children and young people	17	2.9	4.0	1.0	[1.5, 0.5]	0.0015
**Provide ongoing support**						
Female victims	18	2.6	3.9	1.5	[2.0, 1.0]	0.0005
Female perpetrators	18	1.6	3.5	2.0	[2.5, 1.5]	0.0002
Male victims	18	2.1	3.8	1.5	[2.0, 1.0]	0.0002
Male perpetrators	18	1.8	3.4	1.5	[2.0, 1.0]	0.0001
Parents	18	2.2	3.6	1.0	[2.0, 1.0]	0.0002
Children and young people	18	2.3	3.6	1.0	[1.5, 1.0]	0.0007

This table reports: the number of paired observations; mean preparedness score [range 1–5] at time points 1 and 2; the Hodges-Lehmann estimate of the median change and its 95% confidence interval (CI); and the Wilcoxon Signed Ranks Test of the change (T2-T1) in median score

These findings were also reflected in interviews in which clinicians indicated increased knowledge and confidence in asking patients about their experiences as a result of the training.

*[T]hat’s been one of the things that’s changed for me from the training, is that it’s part of my standard routine for people with depressive or panic attack type symptoms particularly, is to include that.* (Clinician3T2)

Interviewed clinicians described ways in which they modified their clinical practice. They reported that the IRIS+ training had raised their awareness about ways DVA may present in general practice and how it may impact on patients’ health.

*[I]t has certainly made me more aware […] of the potential of patients who are either victims, mainly victims, but also perpetrators of domestic abuse, what kind of other issues they may present with. […] alcohol and mental health issues being the more obvious things but also a variety of other more physical health issues. Other more subtle ways in which patients may present. It is sometimes worth just exploring that a little bit in consultations.* (Clinician2T1)

Training encouraged clinicians to have a low threshold for asking about DVA, as well as giving them alternative ways to ask.

*‘Does it ever get scary?’ - I have actually used that -* mentioned a doctor (Clinician8T1).

Interviewed seven months after completing the training sessions, one GP explained how he had altered his ways of exploring the presence of DVA:

*I wasn’t asking as directly about domestic violence before. Clearly, if someone was beaten up, I’d ask. Obviously, you do that. But not necessarily, say, implementing that in with depression. Whereas, as a result, since, I have actually directly asked people. […] I would now specifically say, ‘Do you get hurt by your partner?’ That’s a change, and I think that’s probably a good result. It’s been quite interesting seeing women - there have been women since - just take that completely in their stride.* (Clinician3T1)

The PIM+ survey demonstrated consistent improvements in clinicians’ self-reported preparedness in referring, recording and supporting female and male adult victims and perpetrators and children affected by DVA nine months after training compared to before training. Learning about safe and appropriate ways of recording DVA in patients’ EMR was seen as a crucial training outcome by clinicians. It has also led to changes in some clinicians’ recording practices.

*That was a key learning for me actually […] How we record things in the notes and how in some cases actually recording might increase the risk for a person. […] It actually led to a change in our practice about how we do that.* (Clinician6T1)

Consistent with the PIM+ survey results, follow-up clinician interviews also demonstrated that clinicians felt substantially more secure in talking to children directly about DVA: *‘[I]f I can talk to them about contraception or something, I can talk to them about domestic violence’* (Clinician9T2) – argued a GP while another spoke about how talking directly to children about DVA had been *‘more at the forefront of my mind’* (Clinician5T2).

In line with post-training improvements in the self-reported confidence and preparadeness in talking to male victims or perpetrators, we have also found some examples of embedded aspects of direct clinical engagement with men about DVA.

*When you have got used to doing it [asking men], it becomes an easier thing to do.* (Clinician2T1) – explained a GP.

Others reported increased self-efficacy due to better awareness of how DVA might affect men*:*

*Since the training, I feel definitely more comfortable broaching things with perpetrators. ‘Do you worry about your behaviour?’ … That sort of stuff […] It is a bit more difficult when they’ve come about other things, like, substance misuse or their mood and then actually asking them, but when the opportunity has arisen, definitely, I’ve got more awareness.* (Clinician6T2).

Clinicians claimed that communication techniques acquired during training had been useful and easy to implement. However, most clinicians found it difficult to intergate these methods into their clinical encounters with male perpetrators.

*It’s easy enough to say to a bloke, ‘The way you’re feeling at the moment, does it boil over into anger and is it causing problems at home?’ I don’t think that would be a particularly difficult thing to ask. […] If someone said that they were drinking a lot, ‘Has it made you violent? Does it make you violent when you drink?’ But I haven’t incorporated these into my routine.* (Clinician3T2)

### Identifications and referrals

Consistent with previous findings [20, 21], all female survivors we interviewed spoke about the meaningful impact of being referred to the IRIS+ hub. The women we talked to said that they felt safe and able to talk about DVA and their home life to clinicians who asked and listened to them in a non-judgemental way. Twenty-eight of the thirty-four women referred to the IRIS+ hub went on to receive direct support from the AE. Referred women were offered trauma-informed, needs-led emotional and practical one to one support on a regular basis for a duration of between one to six months (Fig. 1. Box 3.1; 3.2)*.*

Women felt that the emotional support received from the AE had made them feel more confident, assertive and less alone. Consistent with previous research about both the initial and longer term benefits of the IRIS style referral [21], our interviews and our questionnaires indicated a reduction of the self-reported number of abusive behaviours experienced by participants and a reduction of the self-reported negative impact of previous abuse on their mental health and wellbeing (Fig. 1. Box 3.1; 3.2; 3.3; 3.5).

*If it wasn’t for IRIS+, I wouldn’t be sitting here with you. […] I saw her [the AE] and I left smiling, I was like, ‘I can fight, I can do it. Today, I’ll smile’.* (Adult7).

The rate of referral of women survivors of DVA (*n* = 33) in the study period was comparable to the original IRIS trial [20] (Fig. 1. Box 2.3; 3.1). The IRIS+ hub also received a substantial number of child referrals alongside their non-abusive parent (*n* = 35), but only one female perpetrator and two male perpetrators were referred to the IRIS+ hub (Table 2.) (Fig. 1. Box 2.3).

**Table 2:** Identification and referral to the IRIS+ hub

Practice	Number of registered patients in 2018	Recorded number of DVA identifications (EMR data)			Recorded number of referrals to the IRIS+ hub		
		Adults		Children	Adults		Children
		**Female**	**Male**		**Female**	**Male**	
Practice A	15700^a^	14	0	1	7	0	2
Practice B	11300^a^	23	1	1	22	2	29
Practice C	10000^a^	4	0	1	2	0	0
Practice D	6500^a^	41	4	46	3	0	4
**Total**		**82**	**5**	**49**	**34**	**2**	**35**
**IRIS+ support offered and accepted**					**28**	**1**	**22** (6 direct support and 16 support for children via mother)

Numbers of recorded identifications of patients experiencing DVA in pilot practices and DVA referrals received by the IRIS+ hub, 10 months after intervention

^a^Rounded to the nearest one hundred

Most referrals were made in the one month period following the training sessions, soon after the brief follow-up IRIS+ session, and in January, following the Christmas holidays. Clinicians other than GPs (nurses, health care assistants, etc.) were difficult to engage with IRIS+ and there was only one referral from a practice nurse (Fig. 1. Box 1.3; 2.3).

Regular e-mail reminders, brief follow-up practice team visits, the availability of the AE to discuss cases, as well as the clinician e-resource were in place during the study period. These, together with feedback from the AE about referred patients to clinicians, facilitated further identifications and referrals. The failure to implement the electronic prompt system linked to the medical record was highlighted as a barrier to DVA identification (Fig. 1. Box 2.1).

Clinicians agreed that IRIS+ takes time to implement and requires continued support to embed in practice. The acceptability and normalisation of directly asking and referring men and children (as well as maintaining already implemented clinical DVA work with women) were seen as requiring continuing skill maintenance, team collaboration, as well as practice leadership and DVA service support.

Variation in referral numbers reflects variation in the clinician engagement with IRIS+ across the four practices. Although all practices referred patients, the majority came from multiple doctors in one practice (Practice B) where most adult identifications resulted in adult and linked children referrals. The intervention’s increased reach in this practice was possibly related to enabling team climate on one hand, and to the identification of an IRIS+ clinical lead within the practice, who actively supported the ongoing implementation of IRIS+ despite other competing demands, on the other. In contrast, area-level factors (high DVA incidence rate in patient population, normalisation of DVA in the community, general suspicion of professional interventions) were identified as being more salient in contributing to a context for very low IRIS+ referral rates in another practice (Practice D).

Our EMR search generated a total 136 patient records with linked DVA codes, free text information and scanned external reports (Table 2.). We found that GPs receive information about patients affected by DVA from other agencies, not just disclosures from patients. One third of identifications were direct disclosures to a clinician during consultations. Two thirds were third party information from reports sent to general practice of which most were police DVA incident reports about families with children or multi-agency risk assessment conference (MARAC) reports about high-risk cases of DVA, but also included letters from A&E, health visitors, and children’s social care. Of those adults experiencing or perpetrating DVA identified in the EMR, 64% were known to have children in the household (Fig. 1. Box 2.2).

The EMR data and the clinician interviews together indicated that there was uncertainty and a considerable variation among practices and individual clinicians about how the practice coded and how clinicians responded to DVA identifications received from other agencies. In Practice D, for example, situated in a deprived sub-urban setting with one of the highest police DVA incident reporting rate per population in the area, direct DVA disclosures to GP constituted 13% of identifications and information from third party constituted 87% of identifications. The equivalent ratio was 42–58% in Practice B, and 67–33% in Practice A. The absence of training and guidance about how to record and respond to third party information [39, 49] contributed to a lack of clarity about expectations and responsibility for action when information about DVA was shared between agencies (Fig. 1. Box 1.3). It also contributed to the difference between the number of patients identified in the EMR (136) and the number referred to the IRIS+ hub (69) (Table 2.).

*Somebody should be coding it appropriately, and it should be part of the record, and accessible as part of the record, because potentially it’s important. And then beyond that, I should be considering it the same way that I consider all the bits of the jigsaw that might be relevant to a particular presentation by a particular patient.* (Clinician9T2)

Most adult patients had a consultation with a doctor following DVA identification by other services. However, the availability and visibility (in the EMR) of these external reports about DVA did not generally prompt GPs to discuss DVA with the patient.

*We have a lot of patients who are recorded that there’s domestic abuse. […] Yes, it’s a vast number. […] We normally deal with the medical issue that they’re presenting with.* […] *Patients would find this a little bit odd if you kept asking them about their domestic violence when they’ve come for something completely different.* (Clinician4T1)

Direct disclosures to GPs were far more likely to result in an IRIS+ referral. 65% of IRIS+ hub referrals followed a direct disclosure to a GP, despite this forming the minority of identifications. The four male victims identified (but not referred) during the study period were all linked to identifications in reports shared with the practice by other agencies.

### Reaching children

Forty-nine children were identified, all but two via third parties, in the EMR as being affected by DVA. Information came from police, MARAC reports and health visitors. Two records were linked to GP consultations with an adult. The EMR search indicated that 31 of the 49 children identified as currently living with DVA had previously experienced DVA and/or been the subject of child protection proceedings or concerns leading to children’s social care involvement. Thirty-five children were referred to the IRIS+ hub, in all cases together with their (non-abusive) mother following a GP consultation (Fig. 1. Box 2.3). Some of these children were not coded in their EMR as experiencing DVA (Fig. 1. Box 1.3; 2.2).

After training, clinicians reported being more confident about talking to children directly (Fig. 1. Box 1.1; 1.2) (Table 1.), yet there was little to no evidence in the parent interviews or the EMR to suggest that clinicians spoke directly to affected children or raised the issue of DVA with parents in the study period (Fig. 1. Box 1.3). This was most concerning in cases where children were presenting with conditions associated with DVA, such as behavioural problems, anxiety or self-harm.

Our interviews with professionals, parents and children identified a series of barriers to children being referred to or receiving support. According to clinicians, consultation time constraints, the lack of clarity about expectations and responsibility for action when information about DVA was shared with general practice deterred these discussions. Clinicians also identified societal perceptions about interventions as a barrier to more effective enquiry and referral (Fig. 1. Box 2.1; 1.3).

*[T]here is a large amount of suspicion in our area, certainly for social services. There are a lot of people with child protection plans. I think probably people don’t make a finer distinction, necessarily, between any kinds of statutory agency. […] Almost the first thing that people say when there’s any suggestion of, well, even depression, people will say, ‘What matters to me most is not having someone thinking about taking my children away’.* (Clinician3T2)

The fear that children could be removed from their care (a commonly identified barrier in previous studies [50, 51]) was also mentioned by parents as a reason for not seeking or accepting support via general practice. Mothers also reported concerns about being negatively judged by professionals or others and concerns that the perpetrator (in many cases the child’s father) might find out about the support, with an attendant potential escalation of risk to themselves and their children. Some mothers said that they did not think their children were affected by the abuse.

The detrimental impact of DVA on their children motivated many mothers to eventually seek support through general practice. A number of interviewed mothers had attended their GP clinic appointment following a trigger event, such as assault, police involvement or family crisis. Others felt unable to cope with chronic severe physical and mental health problems, isolation or the pressures of parenting.

*I tried to take an overdose. I just couldn’t cope anymore. I knew I had to do something because of the children. I’m the only thing they’ve got. So that was a big push for me to see the GP and to tell him what had happened.* (Adult12)

Twenty-two children and young people received IRIS+ hub support either directly from the CYPW (*n* = 6) or indirectly via their mother (*n* = 16). Mothers of eleven children declined the offer to receive support. Direct support was individually tailored to the child and included one to one sessions providing a wide range of emotional and practical support and might have also included participation in a group programme for young people about healthy relationships. Support for parents around children included parenting advice, legal advice around child contact and resident arrangements, onward service referrals, support with access to safe accommodation, childcare or parent/child activity sessions (Fig. 1. Box 3.1; 3.2).

Interviewed parents said that their children benefited from the IRIS+ support. Mothers reported that both the direct and indirect support improved family relationships and led to improved physical and mental health, wellbeing and confidence of their children (Fig. 1. Box 3.4; 3.5; 4.1; 4.2; 4.3). A study participant, who also received extensive support from the AE herself, attributed the improvement in her teenage child to IRIS+ support:

*The wrist-cutting stopped completely. She [CYPW] talked to her […] Before she would always say, ‘I don’t want to live.’ She said no-one loved her, no-one wanted her... Now it’s not like that. She no longer harms herself.* (Adult6)

Children voiced their appreciation for the support they had been given. As one young person summed up her experience with the intervention:

*Just have a chat, just have adult conversations with someone in an environment that isn’t home. Yes, just having that safe space that I can talk about stuff and know that I’m going to be getting quite personal advice because she [CYPW] knew everything. […] It felt like there was always someone there for us.* (Child3)

### Not reaching men

The PIM+ survey and interviews with clinicians demonstrated post-training improvements in self-reported preparedness to identify and respond to men affected by DVA. Data also showed that discussing DVA with men, offering them a referral to the IRIS+ hub and providing ongoing care for male victims and perpetrators were seen as reasonably compatible with participating clinicians’ roles (Fig. 1. Box 1.1; 1.2).

*We do see chaps that have got anger issues... I am definitely asking more about ‘Do you think anyone at home is scared of your behaviour?’ or ‘Do you think you frighten anyone?’, those sort of less confrontational questions […] whereas before I probably wouldn’t, other than maybe checking if there are children in the house or whether they’ve been violent. I wouldn’t have thought about the overall picture of domestic violence. […] I think I am going to try and push more with people that do come with mood swings, and similar things.* (Clinician6T2*)*

However, increased knowledge and awareness and the direct referral pathway to the AE proved insufficient to trigger identification and referral of men (Fig. 1. Box 1.3; 2.2; 2.3). There were only five male patients (four victims, one perpetrator) identified in the EMR with historic or current forms of DVA during the study period. All five records were linked to information sent to general practice from other agencies (police, hospital, MARAC reports) and coded by the practice as DVA. We found no evidence in the EMR for any diccussion about DVA taking place or a referral being offered to affected male patients. The IRIS+ hub had received only two male perpetrator and no male victim referrals. One referred man had accepted support and took part in the group perpetrator intervention. None of the referred men consented to a research interview.

The interviews with clinicians shed light on specific barriers to referring men to the IRIS+ hub (Fig. 1. Box 1.3; 2.1; 3.1; 3.2). These included relatively low prevalence of male survivors compared to female survivors in the general population; less contact with men due to infrequent GP visits compared to women; assumption that men are unwilling to disclose due to concerns about confidentiality; clinicians’ anxiety about having difficult conversations with individuals who might be violent: ‘*You wonder about asking them a slightly inflammatory question... whether that would set them off’* (Clinician6T2); and lack of confidence asking male patients about DVA when they present with unrelated symptoms: *‘It’s probably simply lack of exposure to perpetrators, really, and to know how their mind-set works, and just making the connection’.* (Clinician3T2).

The lack of evidence about the effectiveness of perpetrator programmes was also mentioned:

*I’m not conscious of having seen any strong evidence that any of these things make a great deal of difference. […] Patients sometimes do, unsurprisingly, ask questions like, ‘Well, what’s the point in me taking this treatment? What will it do? What potentially have I got to gain from this?’ and if you can’t answer that question, then it puts you in a difficult situation.* (Clinician9T1)

Clinicians also identified wider cultural, social and attitudinal barriers to referring men. These included prevalent stereotypes about masculinity, shame associated with being a male victim, normalisation of violence in marginalised communities, and a general mistrust/suspicion of health and social care professionals due to fear of authority or previous negative experience. Clinicians working in a practice with high levels of reported DVA incidents said that domestic abuse was broadly accepted in the local community as a ‘normal’ part of family life:

*My gut feeling is that it’s probably one of those things that’s accepted, like drink driving was and like not wearing your seatbelt was.* (Clinician4T1)

The time pressure within individual consultations was also identified as a barrier to asking male patients about DVA:

*Certainly, there were some of the GPs in the training who were on board and I think who really would have been doing their best to identify those male perpetrators, but a lot of the GPs found that very difficult, and those barriers, I think, were just insurmountable. It was just too big of an ask to be able to ask those questions in that timeframe.* (IRIS+ AE1)

The lack of direct identification and the low number of referrals suggest that clinicians and male patients were unable to overcome the above barriers during the study period. We could not gain insights directly from male patients about the factors that might have inhibited DVA disclosures or the factors that blocked the adaptation of existing effective general practice responses to men affected by DVA (Fig. 1. Box 3.1; 3.2; 3.3; 3.4; 3.5). Our findings nonetheless indicate that the IRIS+ intervention, in its tested form and timeframe, was not feasible in terms of identifying and engaging male victims and perpetrators through general practice.

## Discussion

Recent policy developments have highlighted general practices’ key role in strengthening the response to all patients affected by DVA regardless of their gender, age, sexuality, or DVA experience [52, 53]. There is, however, uncertainty about the value and effectiveness of integrated programmes. The IRIS+ study developed and tested the acceptability and feasibility of an integrated evidence-based primary care system-level DVA intervention.

### Clinician engagement

The IRIS+ training and support programme was highly rated by general practice clinicians and service provider professionals participating in the study. This is a rare finding in contemporary primary care research given the current workload pressures on general practices [54].

While the intervention was popular among all participating clinicians, there was a large variation in both the level of clinician engagement with IRIS+ and the rates of referrals across the four practices. This variation is in line with that shown in other studies [55, 56], including recent findings from the IRIS implementation evaluation [57].

### Identifications and referrals

Our findings around the identification and referral of female survivors resonate with previous research [20, 21] demonstrating that training and support programmes can improve the response of primary care clinicians to women experiencing abuse.

Despite the association between perpetrating or experiencing negative behaviours consistent with DVA and mental health problems in men [16, 42, 43], previous evidence also indicates a discrepancy between headline prevalence figures for male victims and the more detailed data on prevalence related to impact [15]. Due to our study design, and in the absence of process and outcome data from men, we can not demonstrate a cause-effect relationship on the barriers to identifications and referrals of affected men. The above discrepancy, however, might be one of the many possible explanations for the low number of male victim identifications in the pilot practices (although it does not explain why the number of male patients identified as perpetrators is also low in our study).

While the number of male referrals were low, IRIS+ trained clinicians referred a substantial number of children for specialist support alongside their non-abusive parent. Our findings suggest that the IRIS+ intervention was acceptable to parents and children, and children benefitted from both direct and indirect support from IRIS+. The identification and referral of children exposed to DVA is a breakthrough finding in this setting and draws attention to the relevance of detecting and responding to DVA among children and young people within general practice. It also suggests that, with suitable training and support, general practice clinicians are well placed and able to appropriately identify and refer children.

Although children were identified and referred by the pilot practices, they were typically referred to the IRIS+ hub with their non-abusive parents. Furthermore, many children, particularly those identified through external agency reports, were not offered a referral to the IRIS+ hub. Third-party reports are important information sources about those affected by DVA, yet, in the absence of uniform guidance about how to use this information, there are uncertainties and variation in practice. The current IRIS+ training intervention did not explicitly address the complex challenge of safely and effectively coding and responding to information received from other agencies.

The analysis of EMR and our sub-study on DVA information sharing [39] both suggest that the notification about DVA from third parties received by GPs has the potential to contribute to better treatment and safeguarding plans of vulnerable patients. Incorporating information received from other agencies into the EMR and using it to inform patient care in the consultation, however, requires careful professional judgement. Training and guidance for GPs about DVA should explicitly address the challenge of recording and responding to information received from other agencies. Furthermore, clinicians should be supported by colleagues with expertise in DVA and safeguarding in this complex area of practice. 

### Implications on feasibility

Clinicians and service providers believed that the IRIS+ hub had filled a service gap and was a valuable resource in identifying and referring male victims and children below child protection service referral thresholds. The IRIS+ training to clinicians, regular reminders, and the availability of a direct referral pathway, were, however, insufficient to catalyse identification and referral of men and direct identification and referral (without their non-abusive parents) of children.

Our study confirms previously identified barriers to men seeking help [25] and to clinicians providing support for men affected by DVA [36, 58]. Findings are also consistent with previous research that highlights barriers to directly identifying and supporting children affected by DVA (child as patient) [28, 45]. Barriers to referring men and directly referring children to IRIS+ proved to be insurmountable during the study period despite increased levels of preparedness and confidence reported by clinicians after training in terms of directly responding to these groups.

The findings indicate that investment in clinical training and resources together with supportive clinical environment and specialist DVA service links enable effective general practice responses. Our study, however, suggests variations in intervention engagement and large inconsistencies in the application of these effective clinical responses. Although the design does not allow us to demonstrate direct cause-effect relationships between contextual factors and implementation, the study highlights the importance of piloting the feasibility of interventions in geographically, socioeconomically and organisationally heterogenous sites.

IRIS+ clinicians and professionals involved in delivering IRIS+ argued that it takes time and extensive initial practice- and system level facilitation as well as ongoing education and support to embed learning about DVA into clinical practice. These findings are in line with learning from process evaluations of previous DVA interventions in the health care context highlighting drivers for intervention sustainability [57, 59]. Akin to other difficult enquiries (e.g. suicidality) [60], it requires repeated efforts before health professionals gain confidence in routinely asking, supporting and referring patients. Feeling more prepared and confident to ask female survivors about DVA was seen as a pre-requisite before engaging with men. None of the pilot practices had previous IRIS training, so every aspect of engagement was new.

We know from the implementation of IRIS [57] that the normalisation of practice responses to female survivors requires repeated training, sustained system-level support and time. Allowing ample time for embedding a step-change in acceptability of asking men and children directly may be needed for the potentially successful activation of this complex area of practice involving other family members. Testing the extent of practice normalisation and sustainability over a more extended period of time was, however, beyond the scope of the current feasibility study. The extension of the healthcare response beyond female survivors of DVA and the identification and referral of men and the *direct* engagement with children were key aims of IRIS+. The very low number of male and direct children identifications and referrals therefore demonstrate that the IRIS+ intervention, in its tested form and timeframe, is not feasible.

### Towards a revised model

Our findings point towards the need for adaptations to and further testing of the IRIS+ model with a special focus on enabling engagement with men who are victims and/or perpetrators (particularly identified in reports received from other agencies). Evidence shows that IRIS and IRIS+ clinicians have been successfully addressing the needs of female survivors and have the knowledge and skills to effectively address the needs of all patients affected by DVA. They are, however, not converting these relevant skillsets into practice responses to men presenting in general practice. A revised model should put more emphasis on the relevance, acceptability and benefits of selectively asking men about DVA [36, 58]. It should also enhance core components of the IRIS+ training to address the specific needs of male patients affected by DVA (victims and perpetrators) representing different types of masculinity and sexuality. The reconfigured intervention should also recognise the many barriers to disclosure that exist for men of all sexualities, including the inadequate support that is available to them outside general practice [25].

A revised model should also focus on strengthening the direct engagement with children exposed to DVA by enhancing relevant third party identification guidance and by extending the training focus on directly supporting children experiencing DVA. Updated IRIS+ training content should cover in more depth the options of advocacy support available for children via the IRIS+ pathway and how clinicians can talk to children and parents about DVA.

The IRIS+ training intervention was primarily designed for core clinical teams working in general practice, including nurses. Nonetheless, our training observations and the referrals indicate that it felt less relevant for practice nurses and nurse practitioners. It also failed to reach other allied health professionals linked with primary care. Both service providers and general practice clinicians stressed the value of widening the professional scope of the intervention to include other key health professionals (e.g. drug and alcohol workers, health visitors, midwifes). Extending the pool of health professionals, training them together, and enabling them to safely record and refer has the potential to increase the identification and referral of men and children. In addition, the identification of and regular communication with a named DVA lead clinician within each practice (as part of the intervention) would potentially generate improved practice engagement with the programme.

### Strengths and limitations

Key strengths of our study are the integration of diverse evidence sources into the development and evaluation of the training intervention and the multi-professional/multi-agency collaborative approach emphasised during the development and feasibility work. Another strength relates to the active involvement of three service user expert groups. Throughout they have provided valuable insights into the perspectives and experiences of survivors and (ex-) perpetrators. The study also benefited from including the views of different professional groups with expertise in DVA and safeguarding and without a specific role in this area, as well as the perspectives of patients, including voices of children.

There are limitations to this study. As the study was testing the feasibility and acceptability of the intervention, it included only a small number of general practices in a single geographical site, although we tried to ensure the diversity of study practices in terms of size, location and population, as well as the diversity of research participants. Another limitation is potential participation bias: the views of clinicians and patients participating in the interview and questionnaire study might reflect the narratives of those individuals who may have been more favourably disposed to the training and/or intervention. The feasibility study design did not aim to assess the effectiveness or cost-effectiveness of the intervention. Moreover, the low number of children research participants and the lack of male participant and female perpetrator research participant voice within the study limited the interpretation of findings. As a result, although the study gives indication of some of the barriers that might prevent people affected from disclosing DVA in general practice, the study did not contribute to our understanding of why some people experiencing or perpetrating DVA do not seek or accept professional support.

## Conclusions

The development and testing of IRIS+ represents an important step towards broadening our understanding of the potential of training and support interventions in primary care settings to adults who are experiencing or perpetrating DVA and their children. It indicates challenges of applicability of the IRIS model (designed to reach female survivors) to the identification and referral of other patient groups, namely to men and children.

The study demonstrates that the IRIS+ intervention is not feasible in the timeframe and form we tested. It also highlights ways in which specific adaptations to the intervention could potentially allow it to progress to a viable model. Further development work is needed to strengthen some specific elements of the training intervention, especially the identification of men, the *direct* engagement with children, and the guidance on responding to information received from other agencies. The feasibility, acceptability, effectiveness and cost-effectiveness of a reconfigured training and advocacy support programme needs to be fully evaluated.

## Supplementary Information

**Additional file 1.** Questionnaire measuring change in clinicians’ preparedness (time point 1).

**Additional file 2.** Questionnaire measuring change in clinicians’ preparedness (time point 2).

**Additional file 3.** Semi-structured interview topic guides for clinicians participating in the training intervention (two time points).

**Additional file 4.** Semi-structured interview topic guides for IRIS+ professionals delivering the training and/or support intervention.

**Additional file 5.** Semi-structured interview topic guides for adult patients referred into and supported by IRIS+ (two time points).

**Additional file 6.** Semi-structured interview topic guides for children and your people completing direct IRIS+ support (two age groups: 8–12 years and 13–18 years).

**Additional file 7.** Questionnaire for adults referred to and supported by IRIS+ eliciting socio-demographic information, information on past and current experiences and impact of DVA, information on physical and mental health, quality of life, use of health services, their children’s health and wellbeing, etc. (two time points).

## Data Availability

Anonymised transcript (as safe and appropriate) and questionnaire data will be stored on the University of Bristol’s Research Data Service Facility. Bona fide researchers will be able to access non-identifiable data upon reasonable request. Access will be subject to a data access agreement and following approval from the REPROVIDE Chief Investigator and the University of Bristol Data Access Committee.

## Abbreviations

AE: Advocate educator
CYPW: Children and young persons’ worker
DVA: Domestic violence and abuse
EMR: Electronic medical records
GP: General practitioner
IRIS: Identification and Referral to Improve Safety
IRIS+: Enhanced Identification and Referral to Improve Safety
MARAC: Multi-agency risk assessment conference
NHS: National Health Service
PIM: PROVIDE Intervention Measure
