# Supplementary material for: Reaching everyone in general practice? Feasibility of an integrated domestic violence training and support intervention in primary care
Source: BMC Fam Pract. 2021 Jan 12;22:19. doi: 10.1186/s12875-020-01297-5 (PMC7802315; doi:10.1186/s12875-020-01297-5)
Supplement: Supplementary file 1 — Additional file 1. Questionnaire measuring change in clinicians’ preparedness (time point 1). [file 12875_2020_1297_MOESM1_ESM.pdf]

Clinician Survey Before IRIS+ Training

IRIS+ study to test the feasibility of a training and support intervention for general practice to improve the response to women, men and children exposed to domestic violence and abuse

Thank you for agreeing to complete this brief baseline survey.

Your participation in this **survey** will help us to **evaluate and improve** the **IRIS+ domestic violence training and support intervention.**

We are asking you to **complete this survey before your IRIS+ training session.** The aim of this survey is to understand where your **baseline** is.

We will then assess the impact of IRIS+ to your daily practice. We are assessing the extent to which the learning form the IRIS+ training will be put into practice as a result of your training. We are also interested to know to what extent the training and support intervention will increase your confidence and preparedness in relation to the management of domestic violence and abuse cases. Remember, we are assessing the effects of the training intervention – not you! We are interested in the average learning and behavioural impact of the training sessions; we are **not concerned with individual answers** and individual results will be **strictly confidential.**

We will not pass on any identifiable information in relation to your responses to your surgery, the trainers or anyone else. We will store information collected in the study in locked filing cabinets and on password protected databases. You can choose to not participate at any time.

Please fill in your answers based on **how confident and prepared you feel today**.

Please answer as honestly as you can.

Please check the box below to confirm your consent to taking part in this study.

☐ I consent to taking part in this research.

Section 1. About You

This section asks for general details about you so that we can understand who has received the training intervention across different practices.

All information will be treated in confidence and anonymised.

Please enter your initials

____

Please enter the DAY of birth

____

What is your age?

◯ 24 or under
◯ 25-29
◯ 30-34
◯ 35-39
◯ 40-44
◯ 45-49
◯ 50-54
◯ 55-59
◯ 60-64
◯ 65-69
◯ 70 or over

What is your gender?

◯ Female
◯ Male
◯ Transgender
◯ Do not identify as female, male, or transgender

What is your job title?

◯ GP ◯ GP trainee ◯ Practice Nurse ◯ Nurse Practitioner ◯ Health Care Assistant
◯ Phlebotomist ◯ Health Visitor ◯ Midwife ◯ Clinical Pharmacist ◯ Drug and alcohol worker
◯ Other

Please specify

____

How many years of practice you have since professional qualification?

◯ 0-3 years ◯ 4-9 years ◯ 10-20 years ◯ 20+ years

For how many years have you worked in a primary health care setting?

◯ 0-3 years ◯ 4-9 years ◯ 10-20 years ◯ 20+ years

How many clinical sessions (half days) do you work per average week in the primary health care setting?

◯ 1 ◯ 2 ◯ 3 ◯ 4 ◯ 5 ◯ 6 ◯ 7 ◯ 8 ◯ 9 ◯ 10

What is the average number of patients you care for (with face-to-face contact) per week in the primary health care setting?

◯ less than 20 patients ◯ 20-39 patients ◯ 40-59 patients ◯ 60-79 patients ◯ 80-99 patients
◯ 100 or more

Do you have a named/designated safeguarding role?

◯ Yes ◯ No

Do you work regularly in any other clinical setting (A&E, prison medicine, homeless medicine, dermatology clinic, etc.)? If so please list here:

____

Section 2. Previous training

This section asks about the training you may have received in relation to addressing domestic violence issues with different types of patient. Although some of the questions are similar it is important for us to know what kind of training you may, or may not, have received.

Throughout this survey, the term domestic violence and abuse or domestic violence is used. This refers to: "any incident or pattern of incidents of controlling, coercive, threatening behaviour, violence or abuse between those aged 16 or over who are, or have been, intimate partners or family members regardless of gender or sexuality. The abuse can encompass, but is not limited to psychological, physical, sexual, financial, emotional." (Cross-government definition of domestic violence and abuse, 2016)

How much previous training about domestic violence and abuse have you had? (Please tick all that apply)

If you have never received domestic violence training please go to Section 3.

- ☐ Watched a video
- ☐ Completed a web-based programme
- ☐ Attended a lecture or talk
- ☐ Attended a skills-based training or workshop
- ☐ Medical/nursing/other school-classroom training
- ☐ Medical/nursing/other school-clinical setting
- ☐ Registrar/other post-graduate training
- ☐ IRIS training
- ☐ HERMES training
- ☐ RESPONDS training
- ☐ Training provided by the Local Children's Safeguarding Board (LSCB)
- ☐ Other in-depth training (more than 4 hours)
- ☐ Other

If Other, please specify

____

Did any of this previous training include information about the following patient groups?

(For the purposes of this questionnaire, a child or young person is anyone aged between 8-16 and an adult anyone aged over 16.)

	Yes	No	Don't know
Female victims	○	○	○
Female perpetrators	○	○	○
Male victims	○	○	○
Male perpetrators	○	○	○
Children's exposure to domestic violence	○	○	○

Section 3. (You are now halfway through the survey)

Please indicate how prepared you feel to perform the following tasks for the different patient groups.

Ask appropriate questions about domestic violence and abuse to:

	Not prepared	Slightly prepared	Moderately prepared	Fairly well prepared	Well prepared
Females who may be victims	○	○	○	○	○
Females who may be perpetrators	○	○	○	○	○
Males who may be victims	○	○	○	○	○
Males who may be perpetrators	○	○	○	○	○
Parents about their children's exposure to domestic violence	○	○	○	○	○
Children or young people	○	○	○	○	○

Identify signs and symptoms associated with domestic violence and abuse for:

	Not prepared	Slightly prepared	Moderately prepared	Fairly well prepared	Well prepared
Females who may be victims	◯	◯	◯	◯	◯
Females who may be perpetrators	◯	◯	◯	◯	◯
Males who may be victims	◯	◯	◯	◯	◯
Males who may be perpetrators	◯	◯	◯	◯	◯
Parents about their children's exposure to domestic violence	◯	◯	◯	◯	◯
Children and young people	◯	◯	◯	◯	◯

Provide appropriate initial response to disclosures about domestic violence and abuse from:

	Not prepared	Slightly prepared	Moderately prepared	Fairly well prepared	Well prepared
Females who may be victims	○	○	○	○	○
Females who may be perpetrators	○	○	○	○	○
Males who may be victims	○	○	○	○	○
Males who may be perpetrators	○	○	○	○	○
Parents about their children's exposure to domestic violence	○	○	○	○	○
Children and young people	○	○	○	○	○

Make appropriate referrals for:

	Not prepared	Slightly prepared	Moderately prepared	Fairly well prepared	Well prepared
Females who may be victims	◯	◯	◯	◯	◯
Females who may be perpetrators	◯	◯	◯	◯	◯
Males who may be victims	◯	◯	◯	◯	◯
Males who may be perpetrators	◯	◯	◯	◯	◯
Parents about their children's exposure to domestic violence	◯	◯	◯	◯	◯
Children and young people	◯	◯	◯	◯	◯

Safely and appropriately record disclosure and suspicion of domestic violence and abuse in medical records for:

	Not prepared	Slightly prepared	Moderately prepared	Fairly well prepared	Well prepared
Females who may be victims	◯	◯	◯	◯	◯
Females who may be perpetrators	◯	◯	◯	◯	◯
Males who may be victims	◯	◯	◯	◯	◯
Males who may be perpetrators	◯	◯	◯	◯	◯
Parents about their children's exposure to domestic violence	◯	◯	◯	◯	◯
Children and young people	◯	◯	◯	◯	◯

Provide ongoing support in my clinical role for:

	Not prepared	Slightly prepared	Moderately prepared	Fairly well prepared	Well prepared
Females who may be victims	○	○	○	○	○
Females who may be perpetrators	○	○	○	○	○
Males who may be victims	○	○	○	○	○
Males who may be perpetrators	○	○	○	○	○
Parents who have children exposed to domestic violence	○	○	○	○	○
Children and young people	○	○	○	○	○

You have now nearly completed the survey. Please answer a final question regarding prevalence.

How many new diagnoses of domestic violence and abuse have you made in the last 6 months for the following patient groups:

	None	1-2	3-5	6-10	11-20	More than 20	N/A
Females who may be victims	○	○	○	○	○	○	○
Females who may be perpetrators	○	○	○	○	○	○	○
Males who may be victims	○	○	○	○	○	○	○
Males who may be perpetrators	○	○	○	○	○	○	○
Parents who have children exposed to domestic violence	○	○	○	○	○	○	○
Children and young people	○	○	○	○	○	○	○

If you have any additional comments, concerns or questions either in relation to the IRIS+ training or this survey, please use the text box below.
You can also email your comments to the research team on iris-plus@bristol.ac.uk

Would you be happy to be contacted by the research team for a brief follow-up telephone interview after training regarding your views on the IRIS+ training intervention?

○ Yes ○ No

Please enter your contact details below so that we can arrange an interview with you. (Your name will not be matched with your survey responses)

Email address

____

Contact telephone number

____
