# Supplementary material for: Reaching everyone in general practice? Feasibility of an integrated domestic violence training and support intervention in primary care
Source: BMC Fam Pract. 2021 Jan 12;22:19. doi: 10.1186/s12875-020-01297-5 (PMC7802315; doi:10.1186/s12875-020-01297-5)
Supplement: Supplementary file 5 — Additional file 5. Semi-structured interview topic guides for adult patients referred into and supported by IRIS+ (two time points). [file 12875_2020_1297_MOESM5_ESM.pdf]

Study to test the feasibility of a training and support intervention for general practice to improve the response to women, men and children exposed to domestic violence and abuse (DVA)

Adult referred IRIS+ clients/patients – baseline

Topic guide*

Introductory statement

Thank you for agreeing to do this interview. Today I would like to ask you some questions about your experiences of seeing the GP [or nurse] and being referred to IRIS+. The interview will last up to an hour. If there are any questions that you don't feel comfortable answering, just tell me and I'll move on to another topic. Or, if you decide you want to stop the interview altogether that's fine, just let me know. Our conversation today is completely confidential.

We are interested in hearing about their views and experiences. Although I have a list of questions, please do mention anything that you think is important that I don't ask you. If anything is unclear during the interview, let me know.

Consent checklist

Check participant:

- Has read the participant information sheet
- Understands that their participation is voluntary and that they can change their mind and withdraw at any time without having to give a reason and this won't impact on the support that they are receiving from the IRIS+ service.
- Understands that if I have serious concerns about their safety, or that of any children they mention, that I may need to share this concern with an appropriate agency.
- Understands that personal information about them (such as my name and address) will be treated with strict confidence and securely stored separately from all other data about them (e.g. interview transcripts).
- Agrees that the anonymised information collected about them (anonymised transcripts) may be used to support the current research and relevant future research, and may be shared anonymously.

Any questions?

Consent for recording

With your permission, I'd like to digitally record the interview. This is so the interview can be transcribed. It will be erased after being transcribed. All names of people or places which might identif y you or others will not be transcribed. Are you happy to continue with the interview and for it to be digitally recorded?

The recording of the interview will be kept securely and only the anonymised transcript of that interview will be used within the research. The recordings themselves will be erased after transcription.

Can I just confirm that I have your consent to be audio-recorded while this interview takes place?

I'll turn the recorder on now then, and for the record state:

Today's date is…… my name is……… and your name is ……… and I have your consent to record this interview? (yes)

Background

- How did you first find out about the study (interview and questionnaire)?
    - (Prompt: did someone tell you about the study – who?)

- Why did you agree to take part in the interview?
    - Did anyone tell you that you should agree to be interviewed
        - (Prompt: Who? Why?)

- If applicable, how did you find filling out the questionnaires?
    - Did you find them difficult or easy to fill in?
    - Did they bring up any difficult feelings or memories?
    - Were there some parts that were especially difficult?

Referral process to IRIS+ and previous support

- Can you recall who it was who talked to you (e.g. GP, Nurse) about your relationship difficulties and the possibility of referral to IRIS+?
- Can you tell me a little bit about what happened during this conversation/visit?
- What made you decide to talk to that [clinician] on that day?
- On that day, what did you think about the [clinician] initial response to your situation?
- Have you seen the same [clinician] before?
- Was it important to you to talk about these difficulties with a [clinician] you knew?
- Have you talked about these difficulties before to this [clinician] or to any other nurse or GP or anyone else at the practice?

- What was the process of being referred to IRIS+ support service like for you?
    - For example, some people have talked of feeling anxious or hopeful about having support? If anxious, what in particular were you worried about?

- Who explained the IRIS+ support service to you? How clearly/well did they explain it?
    - E.g. was anything confusing?
    - Would anything else have helped you to understand it more clearly?
    - Did you feel able to ask questions? Why? Why not?

- How would you have felt about contacting IRIS+ support worker (or any other support agency) yourself if the clinician had not referred you?
    - Would you have done it?
    - Would you have referred yourself if they had given you a card with a number?
    - If they had shown you a website with details?
    - If didn't contact support agency, why didn't you want to take this up?
- Have you talked about these difficulties/issues to do with your relationships before to a nurse or GP or anyone else at the practice?
    - Who else had you talked to?
        - Why had you felt able to talk to this person/people? How did they respond?
    - How openly were you able to talk about these issues?
        - What helped/stopped you talking about these issues?
    - Ever talked to family or friends about these issues?
        - How did they respond?

- Could anyone have helped you and your family earlier?

- Were there missed opportunities for help do you think?

Work with IRIS+ support worker

- Have you already met the IRIS+ worker? (How many times?)
- Where did you meet them?
  - Is this a good location for you?
  - Has travelling to the location been acceptable?

- How often do you think you are you going to see the IRIS+ support worker? /How often would you like to see the IRIS+ support worker?

- Has your partner/child (if appropriate) been aware of you meeting with the IRIS+ support worker? How do you feel about this/what do they think?

- How are you getting on so far? Does s/he feel like someone you can trust? What makes you feel this way (if does/does not trust)?

- Thinking back to before you met your support worker:
  - What did you hope the support worker could help you with?
  - Do you think that they are helping you with this? How? Why/why not?

Final points

- Thinking about your experiences so far of being referred to and supported by IRIS+, are there any ways you think the support from the [primary healthcare professional you saw] or IRIS+ support worker could be improved?

- Is there anything else you think is important for us to know about your experiences of being involved in the study?

- Are you happy for the recording of the interview to be kept?

- Thank you for your time. Explain that will contact them again for follow up interview, probably around [X time].

Adult IRIS+ clients/patients – follow up

Topic guide*

Introductory statement

Consent checklist

Consent for recording

Work with IRIS+

- How often have you seen/do you see your IRIS+ support worker?

- Where do you meet them?
    - Is this a good location for you?
    - Has travelling to the location been acceptable?

- Has your partner/child (if appropriate) been aware of you meeting the IRIS+ support worker?

- How have you been getting on since we last spoke in [month]? Does s/he feel like someone you can trust?

- Thinking back to before you met your support worker:
    - You told me when we last spoke that you hoped that your support worker would be able to help you with [insert text]?
    - Has your support worker been able to help you with these things?
    - Has your support worker been able to help you with anything else?

- What have you found most useful about having an IRIS+ support worker? Can you give examples?

- What have you found most difficult/challenging about having an IRIS+ support worker? Can you give examples?

- If there are children – have they been offered support?
    - If yes – what type of support and from whom?
    - Was this taken up? Why? / Why not? How useful has it been?
    - If not offered – would it have been useful? Why?
    - Have you tried before to get help for your children?
    - From whom?
    - What was the reaction?

- Are there any ways you think the support from IRIS+ could be improved?

Trajectory since referral to IRIS+

- How would you describe your relationship with your partner / ex-partner and your family [children?] before you had an IRIS+ support worker?
    - Have those relationships changed at all since you have had a support worker?
    - How would you describe them now?
    - How do you think you ex/partner / family/ children would describe your relationship both in the past and now?
    - [If changed – why do you think this is?]

- Has anything else significant changed in your life [in the time period of having a referral to IRIS+ that has affected your relationships?

Final points

- Do you have a strategy for getting support in the future if things get difficult in your relationships?
    - What do you think you will do / who will you talk to?

- Is there anything else you think is important for us to know about your experiences of being involved in the study?

- Are you happy for the recording of the interview to be kept?

- Thank you for your time.

*Please note this is a suggested guide for interviews only. In keeping with standard practice in qualitative research, these questions may be modified & added to as the study progresses and new themes or areas of interest emerge. We will also adapt this guide for use with participants at earlier or later stages of the study.
