# Supplementary material for: Reaching everyone in general practice? Feasibility of an integrated domestic violence training and support intervention in primary care
Source: BMC Fam Pract. 2021 Jan 12;22:19. doi: 10.1186/s12875-020-01297-5 (PMC7802315; doi:10.1186/s12875-020-01297-5)
Supplement: Supplementary file 7 — Additional file 7. Questionnaire for adults referred to and supported by IRIS+ eliciting socio-demographic information, information on past and current experiences and impact of DVA, information on physical and mental health, quality of life, use of health services, their children’s health and wellbeing, etc. (two time points). [file 12875_2020_1297_MOESM7_ESM.pdf]

Workstream 1 IRIS+ – FORM A

Baseline

CONFIDENTIAL

This questionnaire asks about your general health and wellbeing as well as about your current and previous experiences of abuse. You may find some of the questions repetitive or distressing. Please be assured that your responses will be kept strictly confidential and will not be seen by anyone outside the study team. Tell the researcher if you find completing the questionnaire too upsetting.

Please complete the questionnaire by yourself and then return it to the researcher who gave it to you.

Office use only

Date Completed: ___ ___ /___ ___ / 20___ ___

Study ID:

Date received and checked by: ___ ___ /___ ___ / 20___ ___

PHQ9 checked: ☐

SAE checked: ☐

Data entered: ___ ___ /___ ___ / 20___ ___

Entered by: ____________________________

THANK YOU FOR TAKING PART IN THIS STUDY

SECTION ONE: About you

Please help us by answering the following questions about yourself.

1. Your age in years:

☐☐

2. Which ethnic group do you belong to? (Please tick the most relevant box below)

White

1☐ English/Welsh/Scottish/Northern Irish/British
2☐ Irish
3☐ Gypsy or Irish Traveller
4☐ Any other White background (please write in) ☐

Mixed / Multiple ethnic groups

1☐ White & Black Caribbean
2☐ White & Black African
3☐ White & Asian
4☐ Any other Mixed /multiple background (please write in) ☐

Asian or Asian British

1☐ Indian
2☐ Pakistani
3☐ Bangladeshi
4☐ Chinese
5☐ Any other Asian background (please write in) ☐

Black /African / Caribbean / Black British

1☐ African
2☐ Caribbean
3☐ Any other Black / African / Caribbean background (please write in) ☐

Other ethnic group

1☐ Arab
2☐ Any other ethnic group, (please write in) ☐

3. How would you describe your sexuality? Please tick one box

1☐ Heterosexual or straight
2☐ Gay or lesbian
3☐ Bisexual
4☐ Other
5☐ Do not know
6☐ Prefer not to say

4. Which of these qualifications do you have?
Please tick ALL the qualifications that apply or, if not specified, the nearest equivalent.

1☐ O-levels, CSEs, GCSEs, O grades, Standard grades.
2☐ NVQ Levels 1-3/GNVQ
3☐ A levels, AS levels, Higher School Certificate
4☐ NVQ levels 4-5, HNC, HND
5☐ Degree or higher degree
6☐ Other qualifications (e.g. City and Guilds, RSA/OCR, BTEC/Edexcel)
7☐ No formal qualifications

5. Which of the following best describes you? (Please tick one box)

1☐ Employed
2☐ Looking after your home/family
3☐ Unemployed and looking for work
4☐ Unable to work due to long term sickness
5☐ Retired from paid work
6☐ In full time education
7☐ Other (please type in) ____________

6. What is your household's total annual income before tax and benefits? (Please tick one box)

1☐ Up to £5,000
2☐ £5,000 up to £11,999
3☐ £12,000 up to £21,999
4☐ £22,000 up to £37,999
5☐ £38,000 up to £71,999
6☐ £72,000 and above
7☐ Prefer not to say/do not know

7. What is your religion? Please tick one box

1☐ No religion
2☐ Christian (including Church of England, Catholic, Protestant and all other Christian denominations)
3☐ Buddhist
4☐ Hindu
5☐ Jewish
6☐ Muslim
7☐ Sikh
8☐ Any other religion (please describe) ____________
9☐ Prefer not to say

8. What is your gender

0☐ Female
1☐ Male
2☐ Transgender
3☐ Other
4☐ Not stated

9. Are you a parent? 1☐ Yes 0☐ No

10. How many people are there in your household including yourself?

☐

11. Details of each household member including children

For each person who lives in your household, could you please list their relationship to you (e.g., partner/ex-partner, children, parents)?	Sex	Age

SECTION TWO: Violence and abuse

We would like to know if you have experienced any of the following BEHAVIOURS from your partner/ex-partner **within** the last 12 months and any time **before** the last 12 months. For each statement listed below, please indicate how often you have experienced this (a) in the last 12 months (left hand side) and (b) before the last 12 months (right hand side). We want to know about all of these experiences, even those you may not have considered very serious.

Sub-section A

Emotional behaviour

1. How often have you experienced any of the following emotional abuse from any partner/ex-partner?

	i. WITHIN the last 12 months			ii. BEFORE the last 12 months		
	Never	Sometimes	Often	Never	Sometimes	Often
a. Isolated from friends or family						
b. Told what to do/not do, where to go/not go, who to see/not see						
c. Made you feel you had to ask permission to do certain things such as going out, seeing friends, etc (above and beyond being polite)						
d. Made you feel afraid by things they did or said						
e. Prevented you from leaving the home						
f. Controlled the family/household money						
g. Threats to hurt you						
h. Extreme jealousy or possessiveness						
i. Told you what to wear or not to wear or how to look						
j. Humiliated/embarrassed you in front of others						

k. Something else relating to emotional behaviour WITHIN the last 12 months (please describe)

l. Something else relating to emotional behaviour BEFORE the last 12 months (please describe)

As well as knowing about your experiences of certain behaviours we want to know if you have engaged in any of these behaviours TOWARDS your partner in the past 12 months and/or before.

2. How often have you done the following to any partner/ ex-partner?

	**i. WITHIN the last 12 months**			**ii. BEFORE the last 12 months**		
	**Never**	**Sometimes**	**Often**	**Never**	**Sometimes**	**Often**
a. Isolated from friends or family						
b. Told partner/ex-partner what to do/not do, where to go/not go, who to see/not see						
c. Made partner/ex-partner feel they had to ask permission to do certain things such as going out, seeing friends, etc (above and beyond being polite)						
d. Made them feel afraid by things you did/said						
e. Prevented partner/ex-partner from leaving the home						
f. Controlled the family/household money						
g. Threats to hurt partner/ex-partner						
h. Extreme jealousy or possessiveness						
i. Told partner/ex-partner what to wear or not to wear or how to look						
j. Humiliated/embarrassed partner/ex-partner in front of others						

k. Something else relating to emotional behaviour WITHIN the last 12 months (please describe)

l. Something else relating to emotional behaviour BEFORE the last 12 months (please describe)

Sub-section B
Physical behaviour

1. **How often have you experienced the following physical abuse from any partner/ex-partner?**

	i. WITHIN the last 12 months			ii. BEFORE the last 12 months		
	Never	Sometimes	Often	Never	Sometimes	Often
a. Slapped/pushed/shoved						
b. Kicked/punched						
c. Beaten up						
d. Burned						
e. Bitten						
f. Restrained/held down/tied up						
g. Put their hands on your throat or neck						
h. Hit with object or weapon						
i. Threatened with object/weapon						
j. Threatened to kill						
k. Prevented you from getting help for injuries						
l. Stalked/followed/harassed you						
m. Locked in house or room						

n. Something else relating to physical behaviour WITHIN the last 12 months (please describe)

o. Something else relating to physical behaviour BEFORE the last 12 months (please describe)

As well as knowing about your experiences of certain behaviours we want to know if you have engaged in any of these behaviours TOWARDS your partner/ex-partner in the past 12 months and/or before.

2. **How often have you done the following to any partner/ ex-partner?**

	**i. WITHIN the last 12 months**			**ii. BEFORE the last 12 months**		
	**Never**	**Sometimes**	**Often**	**Never**	**Sometimes**	**Often**
a. Slapped/pushed/shoved						
b. Kicked/punched						
c. Beaten up						
d. Burned						
e. Bitten						
f. Restrained/held them down/tied them up						
g. Put your hands on their throat or neck (trying to choke or strangle or suffocate)						
h. Hit with object or weapon						
i. Threatened with object/weapon						
j. Threatened to kill						
k. Prevented them from getting help for injuries						
l. Stalked/followed/harassed them						
m. Locked them in house or room						

n. Something else relating to physical behaviour WITHIN the last 12 months (please describe)

o. Something else relating to physical behaviour BEFORE the last 12 months (please describe)

Sub-section C
Sexual behaviour

1. **How often have you experienced the following sexual abuse from any partner/ex-partner?**

	i. WITHIN the last 12 months			ii. BEFORE the last 12 months		
	Never	Sometimes	Often	Never	Sometimes	Often
a. Touched in a way which caused fear/alarm/distress						
b. Forced into doing something sexual you didn't want to						
c. Hurt during sex						
d. Had boundaries or safe words disrespected						
e. Made to have sex when you didn't want to or didn't stop when you wanted to						
f. Sexually assaulted or abused in any way						
g. Threats to sexually assault/abuse you						

h. Something else relating to sexual behaviour WITHIN the last 12 months (please describe)

i. Something else relating to sexual behaviour BEFORE the last 12 months (please describe)

As well as knowing about your experiences of certain behaviours we want to know if you have engaged in any of these behaviours TOWARDS your partner/ex-partner in the past 12 months and/or before.

2. How often have you done the following to any partner/ ex-partner?

	i. WITHIN the last 12 months			ii. Before the last 12 months		
	Never	Sometimes	Often	Never	Sometimes	Often
a. Touched in a way which caused fear/alarm/distress						
b. Forced into doing something sexual they didn't want to						
c. Hurt during sex						
d. Disrespected boundaries or safe words						
e. Made them have sex when they didn't want to or didn't stop when they wanted you to						
f. Sexually assaulted or abused them in any way						
g. Threats to sexually assault/abuse						

h. Something else relating to sexual behaviour WITHIN the last 12 months (please describe)

i. Something else relating to sexual behaviour BEFORE the last 12 months (please describe)

Sub section D

Impact of abusive behaviour you have experienced on you

1. **Please tick all of the ways any of the above behaviour has affected you.**

1☐ Injuries such as bruises/scratches/minor cuts

2☐ Injuries needing help from doctor/hospital

3☐ Didn’t have an impact

4☐ Lost respect for your partner/ex-partner

5☐ Made you want to leave partner/ ex-partner

6☐ Depression/sleeping problems

7☐ Stopped trusting partner/ex-partner

8☐ Felt unable to cope

9☐ Felt worthless or lost confidence

10☐ Felt sadness

11☐ Felt anxious/panic/lost concentration

12☐ Felt isolated/stopped going out

13☐ Felt angry/shocked

14☐ Self-harmed/felt suicidal

15☐ Worried partner/ex-partner might leave

16☐ Defended self/children/pets

17☐ Feared for life

18☐ Felt had to watch what you say/do

19☐ Not applicable

20☐ Something else relating to the impact of the abusive behaviour – please say what

2. **If you answered questions above about behaving in an abusive way towards your partner/ex-partner what impact do you think your behaviour had on your partner/ex-partner?**

1☐ Injuries such as bruises/scratches/minor cuts

2☐ Injuries needing help from doctor/hospital

3☐ Didn't have an impact

4☐ They lost respect for you

5☐ Made them want to leave you

6☐ Depression/Sleeping problems

7☐ They stopped trusting you

8☐ They felt unable to cope

9☐ Felt worthless or lost confidence

10☐ Felt sadness

11☐ Felt anxious/panic/lost concentration

12☐ Felt isolated/stopped going out

13☐ Felt angry/shocked

14☐ Self-harmed/felt suicidal

15☐ Feared for their life

16☐ They had to be careful of what they said/did

17☐ Not applicable

18 ☐ Something else relating to the impact of the abusive behaviour – please say what

3. **Why do you think you did any of these things:**

Please tick all that apply.

1☐ To stop them from doing something

2☐ Made you feel in control

3☐ Because they were laughing at you

4☐ Because they betrayed/rejected you

5☐ To make them do something you wanted them to do

6☐ Because you didn't trust them

7☐ Because of your alcohol/drug use

8☐ To stop them from leaving you

9☐ Didn't feel good enough/felt insecure

10☐ Because you were jealous/possessive

11☐ Not applicable

12☐ Some other reason – please say what

4.In the last twelve months, how often have the police been called to your house because of violence/abuse your partner/ex-partner was using?

1☐Not at all 2☐Once 3☐2-5 times 4☐6-10 times 5☐More than 10 times

Sub section E

Questions relating to your childhood experience:

Now we want to ask about how your parents (or your parent and their boyfriend/girlfriend/partner) sorted out disagreements. Please **tick** 'Yes' or 'No' for each question.

**1. At any time in your life did:**	**Yes**	**No**
a) You **SEE** a parent get **pushed, slapped, hit, punched, or beaten up** by another parent or their boyfriend or girlfriend?	1☐	0☐
b) One of your parents **threaten to hurt** another parent and it seemed they might really get hurt?	1☐	0☐
c) One of your parents, because of an argument, break or ruin anything belonging to another parent, punch the wall, or throw something?	1☐	0☐
d) One parent get **kicked, choked or beaten up** by your other parent?	1☐	0☐
**2. Before the age of 16 have you ever….**	**Yes**	**No**
a) Been beaten by a parent, step-parent, relative or carer on one or more occasions?	1☐	0☐
b) Been made to do something sexual, such as touching, groping or removing clothes?	1☐	0☐
c) Had unwanted sexual intercourse?	1☐	0☐
d) Been taken into care?	1☐	0☐

SECTION THREE: Your health and well-being

Sub-section A

1. Do you have any current or past mental health problems? (Please include any diagnosis you have been given)

1☐ Yes 0☐ No

If your answer is yes, please describe

2. Have you had any treatment or support for these current or past mental health problems?

1☐ Yes 0☐ No

If your answer is yes, please describe

Sub-section B

This survey asks for your views about your health. This information will help keep track of how you feel and how well you are able to do your usual activities. Answer each question by selecting the answer as indicated. If you are unsure how to answer a question, please give the best answer you can.

	**Excellent**	**Very good**	**Good**	**Fair**	**Poor**
1. In general, would you say your health is	1☐	2☐	3☐	4☐	5☐

2. The following questions are about activities you might do during a typical day. Does your health now limit you in these activities? If so, how much?

	**Yes, limited a lot**	**Yes, limited a little**	**No, not limited at all**
a. Moderate activities such as moving a table, pushing a vacuum cleaner, bowling or playing golf	1☐	2☐	3☐
b. Climbing several flights of stairs	1☐	2☐	3☐

3. During the **past 4 weeks**, how much of the time have you had any of the following problems with your work or other regular daily activities as a result of your physical health?

	**All of the time**	**Most of the time**	**Some of the time**	**A little of the time**	**None of the time**
a. Accomplished less than you would like	1☐	2☐	3☐	4☐	5☐
b. Were limited in the kind of work or other activities	1☐	2☐	3☐	4☐	5☐

4. During the **past 4 weeks**, how much of the time have you had any of the following problems with your work or other regular daily activities as a result of any emotional problems (such as feeling depressed or anxious)?

	**All of the time**	**Most of the time**	**Some of the time**	**A little of the time**	**None of the time**
a. Accomplished less than you would like	1☐	2☐	3☐	4☐	5☐
b. Did work or activities less carefully than usual	1☐	2☐	3☐	4☐	5☐

5. During the past **4 weeks**, how much did pain interfere with your normal work (including both work outside the home and housework)?

Not at all	A little bit	Moderately	Quite a bit	Extremely
1☐	2☐	3☐	4☐	5☐

6. These questions are about how you feel and how things have been with you during the **past 4 weeks**. For each question, please give the one answer that comes closest to the way you have been feeling. How much of the time **during the past 4 weeks**…

	All of the time	Most of the time	Some of the time	A little of the time	None of the time
a. Have you felt calm and peaceful?	1☐	2☐	3☐	4☐	5☐
b. Did you have a lot of energy?	1☐	2☐	3☐	4☐	5☐
c. Have you felt downhearted and depressed?	1☐	2☐	3☐	4☐	5☐

7. During the **past 4 weeks**, how much of the time has your physical health or emotional problems interfered with your social activities (like visiting friends, relatives etc.)?

All of the time	Most of the time	Some of the time	A little of the time	None of the time
1☐	2☐	3☐	4☐	5☐

Sub-section C

Under each heading, please tick the ONE box that best describes your health TODAY.

1. MOBILITY

1☐ I have no problems in walking about

2☐ I have slight problems in walking about

3☐ I have moderate problems in walking about

4☐ I have severe problems in walking about

5☐ I am unable to walk about

2. SELF-CARE

1☐ I have no problems washing or dressing myself

2☐ I have slight problems washing or dressing myself

3☐ I have moderate problems washing or dressing myself

4☐ I have severe problems washing or dressing myself

5☐ I am unable to wash or dress myself

3. USUAL ACTIVITIES (*e.g. work, study, housework, family or leisure activities)*

1☐ I have no problems doing my usual activities

2☐ I have slight problems doing my usual activities

3☐ I have moderate problems doing my usual activities

4☐ I have severe problems doing my usual activities

5☐ I am unable to do my usual activities

4. PAIN/DISCOMFORT

1☐ I have no pain or discomfort

2☐ I have slight pain or discomfort

3☐ I have moderate pain or discomfort

4☐ I have severe pain or discomfort

5☐ I have extreme pain or discomfort

5. ANXIETY/DEPRESSION

1☐ I am not anxious or depressed

2☐ I am slightly anxious or depressed

3☐ I am moderately anxious or depressed

4☐ I am severely anxious or depressed

5☐ I am extremely anxious or depressed

6.

- We would like to know how good or bad your health is TODAY.
- This scale is numbered 0 to 100.
- 100 means the best health you can imagine.
- 0 means the worst health you can imagine.
- Mark an X on the scale to indicate how your health is TODAY.
- Now, please write the number you marked on the scale in the box below.

Your health today =

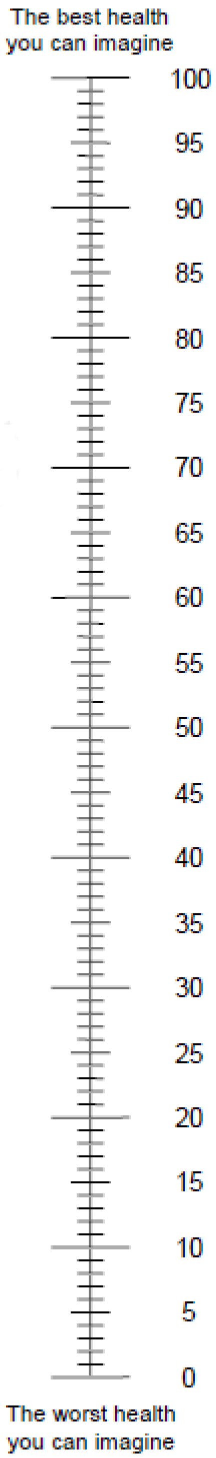

Sub-section D

1. **Over the last 2 weeks, how often have you been bothered by any of the following problems? Please tick the response that best describes you.**

	Not at all	Several days	More than half the days	Nearly every day
a) Little interest or pleasure in doing things?	0☐	1☐	2☐	3☐
b) Feeling down, depressed, or hopeless?	0☐	1☐	2☐	3☐
c) Trouble falling or staying asleep, or sleeping too much?	0☐	1☐	2☐	3☐
d) Feeling tired or having little energy?	0☐	1☐	2☐	3☐
e) Poor appetite or overeating?	0☐	1☐	2☐	3☐
f) Feeling bad about yourself - or that you're a failure or have let yourself or your family down?	0☐	1☐	2☐	3☐
g) Trouble concentrating on things, such as reading the newspaper or watching television?	0☐	1☐	2☐	3☐
h) Moving or speaking so slowly that other people could have noticed? Or the opposite - being so fidgety or restless that you have been moving around a lot more than usual?	0☐	1☐	2☐	3☐
i) Thoughts that you would be better off dead, or of hurting yourself in some way?	0☐	1☐	2☐	3☐

j. If you have ticked any problems, how difficult have these problems made it for you to do work, take care of things at home, or get along with people?

0☐ Not difficult at all 1☐ Somewhat difficult 2☐ Very difficult 3☐ Extremely difficult

Sub-section E

1. <u>Over the last 2 weeks</u>, how often have you been bothered by any of the following problems? Please tick the response that best describes you.

	**Not at all**	**Several days**	**Over half the days**	**Nearly every day**
a) Feeling nervous, anxious, or on edge?	0☐	1☐	2☐	3☐
b) Not being able to stop or control worrying?	0☐	1☐	2☐	3☐
c) Worrying too much about different things?	0☐	1☐	2☐	3☐
d) Trouble relaxing?	0☐	1☐	2☐	3☐
e) Being so restless that it is hard to sit still?	0☐	1☐	2☐	3☐
f) 6. Becoming easily annoyed or irritable?	0☐	1☐	2☐	3☐
g) 7. Feeling afraid as if something awful might happen?	0☐	1☐	2☐	3☐

h) If you checked off any problems, how difficult have these made it for you to do your work, take care of things at home, or get along with other people?

☐ Not difficult at all ☐ Somewhat difficult ☐ Very difficult ☐ Extremely difficult

<u>Sub-section F</u>

Below is a list of problems that people sometimes have in response to a very stressful experience. Please read each problem carefully and then tick one of the boxes to the right to indicate how much you have been bothered by that problem <u>in the past month</u>.

1. <u>In the past month</u>, how much were you bothered by:

	Not at all	A little bit	Moderately	Quite a bit	Extremely
a) Repeated, disturbing, and unwanted memories of the stressful experience?	0☐	1☐	2☐	3☐	4☐
b) Repeated, disturbing dreams of the stressful experience?	0☐	1☐	2☐	3☐	4☐
c) Suddenly feeling or acting as if the stressful experience were actually happening again (as if you were actually back there reliving it)?	0☐	1☐	2☐	3☐	4☐
d) Feeling very upset when something reminded you of the stressful experience?	0☐	1☐	2☐	3☐	4☐
e) Having strong physical reactions when something reminded you of the stressful experience (for example, heart pounding, trouble breathing, sweating)?	0☐	1☐	2☐	3☐	4☐
f) Avoiding memories, thoughts, or feelings related to the stressful experience?	0☐	1☐	2☐	3☐	4☐
g) Avoiding external reminders of the stressful experience (for example, people, places, conversations, activities, objects, or situations)?	0☐	1☐	2☐	3☐	4☐
h) Trouble remembering important parts of the stressful experience?	0☐	1☐	2☐	3☐	4☐
i) Having strong negative beliefs about yourself, other people, or the world (for example, having thoughts such as: I am bad, there is something seriously wrong with me, no one can be trusted, the world is completely dangerous)?	0☐	1☐	2☐	3☐	4☐
j) Blaming yourself or someone else for the stressful experience or what happened after it?	0☐	1☐	2☐	3☐	4☐

	Not at all	A little bit	Moderately	Quite a bit	Extremely
k) Having strong negative feelings such as fear, horror, anger, guilt, or shame?	0☐	1☐	2☐	3☐	4☐
l) Loss of interest in activities that you used to enjoy?	0☐	1☐	2☐	3☐	4☐
m) Feeling distant or cut off from other people?	0☐	1☐	2☐	3☐	4☐
n) Trouble experiencing positive feelings (for example, being unable to feel happiness or have loving feelings for people close to you)?	0☐	1☐	2☐	3☐	4☐
o) Irritable behaviour, angry outbursts, or acting aggressively?	0☐	1☐	2☐	3☐	4☐
p) Taking too many risks or doing things that could cause you harm?	0☐	1☐	2☐	3☐	4☐
q) Being “superalert” or watchful or on guard?	0☐	1☐	2☐	3☐	4☐
r) Feeling jumpy or easily startled?	0☐	1☐	2☐	3☐	4☐
s) Having difficulty concentrating?	0☐	1☐	2☐	3☐	4☐
t) Trouble falling or staying asleep?	0☐	1☐	2☐	3☐	4☐

Sub-section G

ABOUT YOUR OVERALL QUALITY OF LIFE

Please indicate which statements best describe your overall quality of life at the moment by placing a tick (✓) in **ONE** box for each of the five groups below.

**1. Feeling settled and secure**		
I am able to feel settled and secure in **all** areas of my life	☐	4
I am able to feel settled and secure in **many** areas of my life	☐	3
I am able to feel settled and secure in **a few** areas of my life	☐	2
I am **unable** to feel settled and secure in **any** areas of my life	☐	1
**2. Love, friendship and support**		
I can have **a lot** of love, friendship and support	☐	4
I can have **quite a lot** of love, friendship and support	☐	3
I can have **a little** love, friendship and support	☐	2
I **cannot** have **any** love, friendship and support	☐	1
**3. Being independent**		
I am able to be **completely** independent	☐	4
I am able to be independent in **many** things	☐	3
I am able to be independent in **a few** things	☐	2
I am **unable** to be at all independent	☐	1
**4. Achievement and progress**		
I can achieve and progress in all aspects of my life	☐	4
I can achieve and progress in **many** aspects of my life	☐	3
I can achieve and progress in **a few** aspects of my life	☐	2
I **cannot** achieve and progress in **any** aspects of my life	☐	1
**5. Enjoyment and pleasure**		
I can have **a lot** of enjoyment and pleasure	☐	4
I can have **quite a lot** of enjoyment and pleasure	☐	3
I can have **a little** enjoyment and pleasure	☐	2
I **cannot** have **any** enjoyment and pleasure	☐	1

Please ensure you have only ticked **ONE** box for each of the five groups.

© 2010 Hareth Al-Janabi and Joanna Coast

Sub-section H

ALCOHOL

This is one unit of alcohol…

…and each of these is more than one unit

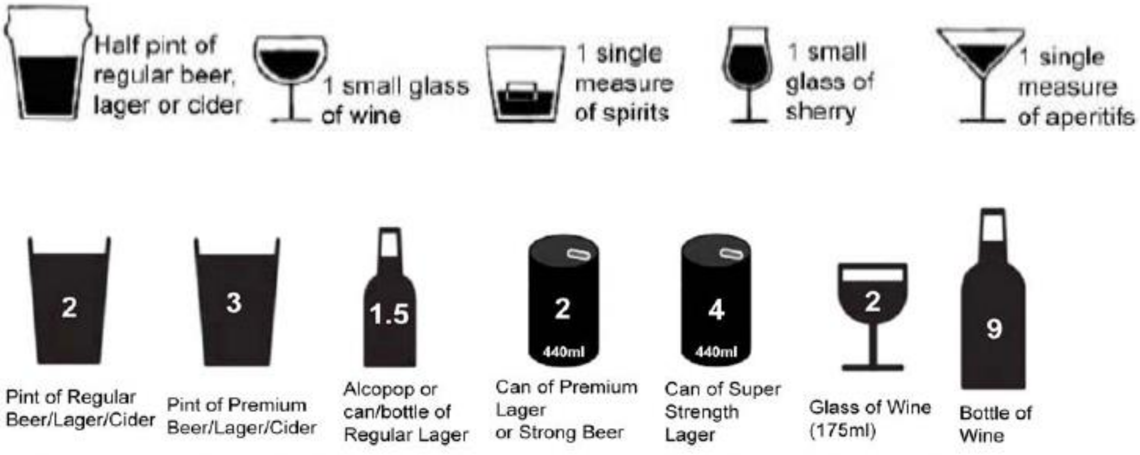

1. How often do you have a drink containing alcohol?

0☐ Never
1☐ Monthly or less
2☐ 2-4 times per month
3☐ 2-3 times per week
4☐ 4+ times per week

2. How many units of alcohol do you drink on a typical day when you are drinking?

0☐ 1 or 2
1☐ 3 or 4
2☐ 5 or 6
3☐ 7-9
4☐ 10+
5☐ N/A

3. How often have you had 6 or more units if female, or 8 or more if male, on a single occasion in the last year?

0☐ Never
1☐ Less than monthly
2☐ Monthly
3☐ Weekly
4☐ Daily or almost daily

4. How often during the last year have you found that you were not able to stop drinking once you had started?

0☐ Never
1☐ Less than monthly
2☐ Monthly
3☐ Weekly
4☐ Daily or almost daily

5. How often during the last year have you failed to do what was normally expected from you because of your drinking?

0☐ Never
1☐ Less than monthly
2☐ Monthly
3☐ Weekly
4☐ Daily or almost daily

6. How often during the last year have you needed an alcoholic drink in the morning to get yourself going after a heavy drinking session?

0☐ Never
1☐ Less than monthly
2☐ Monthly
3☐ Weekly
4☐ Daily or almost daily

7. How often during the last year have you had a feeling of guilt or remorse after drinking?

0☐ Never
1☐ Less than monthly
2☐ Monthly
3☐ Weekly
4☐ Daily or almost daily

8. How often during the last year have you been unable to remember what happened the night before because you had been drinking?

0☐ Never
1☐ Less than monthly
2☐ Monthly
3☐ Weekly
4☐ Daily or almost daily

9. Have you or somebody else been injured as a result of your drinking?

No	Yes, but not in the last year	Yes, during the last year
0☐	2☐	4☐

10. Has a relative or friend, doctor or other health worker been concerned about your drinking or suggested that you cut down?

No	Yes, but not in the last year	Yes, during the last year
0☐	2☐	4☐

Sub-section I

DRUGS

Here are a few questions about drugs. Please answer as correctly and honestly as possible by indicating which answer is right for you.
If you are not sure what we mean by drugs there is a separate sheet which explains what is and isn't a drug. We are also not including medications prescribed by a doctor if they have been prescribed for you and you are using them as prescribed.

1. How often do you use drugs other than alcohol?	**Never**	**Once a month or less often**	**2-4 times a month**	**2-3 times a week**	**4 times a week or more often**
2. Do you use more than one type of drug on the same occasion?	**Never**	**Once a month or less often**	**2-4 times a month**	**2-3 times a week**	**4 times a week or more often**
3. How many times do you take drugs on a typical day when you use drugs?	**0**	**1-2**	**3-4**	**5-6**	**7 or more**
4. How often are you influenced heavily by drugs?	**Never**	**Less often than once a month**	**Every month**	**Every week**	**Daily or almost every day**
5. Over the past year, have you felt that your longing for drugs was so strong that you could not resist it?	**Never**	**Less often than once a month**	**Every month**	**Every week**	**Daily or almost every day**
6. Has it happened, over the past year, that you have not been able to stop taking drugs once you started?	**Never**	**Less often than once a month**	**Every month**	**Every week**	**Daily or almost every day**
7. How often over the past year have you taken drugs and then neglected to do something you should have done?	**Never**	**Less often than once a month**	**Every month**	**Every week**	**Daily or almost every day**
8. How often over the past year have you needed to take a drug the morning after heavy drug use the day before?	**Never**	**Less often than once a month**	**Every month**	**Every week**	**Daily or almost every day**
9. How often over the past year have you had guilt feelings or a bad conscience because you used drugs?	**Never**	**Less often than once a month**	**Every month**	**Every week**	**Daily or almost every day**
10. Have you or anyone else been hurt (mentally or physically) because you used drugs?	No		**Yes, but not over the past year**		**Yes, over the past year**
11. Has a relative or a friend, a doctor or a nurse, or anyone else, been worried about your drug use or said to you that you should stop using drugs?	No		**Yes, but not over the past year**		**Yes, over the past year**

Sub-section J

GAMBLING

1. In the last 12 months, have you spent any money on gambling activities? This includes purchasing lottery tickets, playing bingo, betting on races or other (e.g., sporting) events, playing virtual gaming machines or slot machines in a bookmaker or any venue, and any form of betting or gambling online?

0☐ No 1☐ Yes

If your answer is yes:

(a) Have there been periods when you spent a lot of time thinking about your gambling experiences or planning out future gambling ventures or bets? 0☐ No 1☐ Yes

(b) Have you tried to stop, cut down or control your gambling? 0☐ No 1☐ Yes

(c) Have you lied to family members, friends, or others about how much you gamble or how much money you lost gambling? 0☐ No 1☐ Yes

SECTION FOUR:

Section A - Your children

1. **Which of the following currently applies to your children?**

Tick all those which apply.

1☐ The courts or state child protection have told my partner/ex-partner they can't live with our children

2☐ The courts or state child protection have stopped me from living with my children

3☐ The courts or state child protection have stopped my partner/ex-partner having contact/access

4☐ The courts or state child protection have stopped me having contact/access

5☐ I have applied to the court for contact with my children

6☐ Partner/ex-partner has applied to the court for contact with our children

7☐ My children have been removed and are being looked after by foster carers

8☐ I don't think my children were affected by the abuse

9☐ My partner/ex-partner doesn't think our children were affected by the abuse

10☐ One or more of my children is angry or upset with me

11☐ One or more of my children is angry/upset with my partner/ex-partner because of what's happened

12☐ One or more of my children is currently registered with the state child protection as in need of protection because of the violence/abuse in my relationship with my partner/ex-partner

13☐ Not Applicable

14☐ Something else in relation to your children

2. **<u>Does your child / any of your children currently have any disabilities or special educational needs?</u>**

☐ Yes ☐ No

3. **<u>If yes, please briefly describe your child's/children's disabilities or special educational needs</u>**

Section B - Your children's health and wellbeing

If you **do not** have any children aged between 8-18 then **please go to section 5**

If you have any **children aged between 8-18,** please fill out the following section thinking about the **child whose birthday is first in the calendar year**.

How is your child? How does she/he feel? This is what we would like to know from you.

Please answer the following questions to the best of your knowledge, ensuring that the answers you give reflect the perspective of the selected child. Please try to remember your child's experiences over the last week...

Are any of your children aged between 8 and 18?

☐ Yes

☐ No ---------------------------------------Go to Section 5

1. **What is your relationship to the child?**

1☐ Mother
2☐ Father
3☐ Stepmother / Father's partner
4☐ Stepfather/mother's partner
5☐ Other
Please state relationship

2. **How old is your child (the selected child)?**

☐☐ years

3. **Is your child (the selected child) female or male?**

0☐ Female
1☐ Male
2☐ If neither of the above please specify your child's preference for gender

Sub-section C

Physical Activities and Health

1. In general, how would your child rate her/his health?

1☐ Excellent
2☐ Very good
3☐ Good
4☐ Fair
5☐ Poor

Thinking about the last week ...

	Not at all	Slightly	Moderately	Very	Extremely
2. Has your child felt fit and well?	1☐	2☐	3☐	4☐	5☐
3. Has your child been physically active (e.g. running, climbing, biking)?	1☐	2☐	3☐	4☐	5☐
4. Has your child been able to run well?	1☐	2☐	3☐	4☐	5☐

Thinking about the last week...

	Never	Seldom	Quite often	Very often	Always
5. Has your child felt full of energy?	1☐	2☐	3☐	4☐	5☐

Sub-section D

General Mood and Your Child's Feelings

Thinking about the last week...

	Not at all	Slightly	Moderately	Very	Extremely
1. Has your child felt that life was enjoyable?	1☐	2☐	3☐	4☐	5☐

Thinking about the last week ...

	Never	Seldom	Quite often	Very often	Always
2. Has your child been in a good mood?	1☐	2☐	3☐	4☐	5☐
3. Has your child had fun?	1☐	2☐	3☐	4☐	5☐

Thinking about the last week ...

	Never	Seldom	Quite often	Very often	Always
4. Has your child felt sad?	1☐	2☐	3☐	4☐	5☐
5. Has your child felt so bad that he/she didn't want to do anything?	1☐	2☐	3☐	4☐	5☐
6. Has your child felt lonely?	1☐	2☐	3☐	4☐	5☐
7. Has your child been happy with the way he/she is?	1☐	2☐	3☐	4☐	5☐

Sub-section E

Family and Your Child's Free Time

Thinking about the last week...

	Never	Seldom	Quite often	Very often	Always
1. Has your child had enough time for him/herself?	1☐	2☐	3☐	4☐	5☐
2. Has your child been able to do the things that he/she wants to do in his/her free time?	1☐	2☐	3☐	4☐	5☐
3. Has your child felt that his/her parent(s) had enough time for him/her?	1☐	2☐	3☐	4☐	5☐
4. Has your child felt that his/her parent(s) treated him/her fairly?	1☐	2☐	3☐	4☐	5☐
5. Has your child been able to talk to his/her parent(s) when he/she wanted to?	1☐	2☐	3☐	4☐	5☐
6. Has your child had enough money to do the same things as his/her friends?	1☐	2☐	3☐	4☐	5☐
7. Has your child felt that he/she had enough money for his/her expenses?	1☐	2☐	3☐	4☐	5☐

Sub-section F

Friends

Thinking about the last week...

	Never	Seldom	Quite often	Very often	Always
1. Has your child spent time with his/her friends?	1☐	2☐	3☐	4☐	5☐
2. Has your child had fun with his/her friends?	1☐	2☐	3☐	4☐	5☐
3. Have your child and his/her friends helped each other?	1☐	2☐	3☐	4☐	5☐
4. Has your child been able to rely on his/her friends?	1☐	2☐	3☐	4☐	5☐

Sub-section G

School and Learning

Thinking about the last week...

	Not at all	Slightly	Moderately	Very	Extremely
1. Has your child been happy at school?	1☐	2☐	3☐	4☐	5☐
2. Has your child got on well at school?	1☐	2☐	3☐	4☐	5☐

Thinking about the last week...

	Never	Seldom	Quite often	Very often	Always
3. Has your child been able to pay attention?	1☐	2☐	3☐	4☐	5☐
4. Has your child got along well with his/her teachers?	1☐	2☐	3☐	4☐	5☐

5.Anything else about your child's health or wellbeing

SECTION 5: Your partner/ex-partner and your relationship

1. At the moment, how often are you fearful of your partner/ex-partner?

1☐ Never   2☐ Not often   3☐ Sometimes   4☐ Often   5☐ Always/mostly

2. What is your relationship status with your partner/ex-partner NOW?

1☐ Together and living together

2☐ Together but living apart

3☐ In the process of splitting up

4☐ The relationship has ended and we are living apart with no contact

5☐ The relationship has ended and we are living apart and still have contact

6☐ I am not sure

7☐ Something else – please say:

3. What are your hopes for your relationship with them in the future?

1☐ That we will be together and living together

2☐ That this relationship will end

3☐ I am not sure

4☐ I am in another relationship already

5☐ Something else – please say what:

THANK YOU FOR COMPLETING THIS QUESTIONNAIRE!

Workstream 1 IRIS+ - Interim

Follow up questionnaire

CONFIDENTIAL

This questionnaire asks about your general health and wellbeing as well as about your current and previous experiences of abuse. You may find some of the questions repetitive or distressing. Please be assured that your responses will be kept strictly confidential and will not be seen by anyone outside the study team. Tell the researcher if you find completing the questionnaire too upsetting.

Please complete the questionnaire by yourself and then return it to the researcher who gave it to you.

Office use only

Date Completed: ___ ___ /___ ___ / 20___ ___

Study ID:

Date received and checked by: ___ ___ /___ ___ / 20___ ___

PHQ9 checked: ☐

SAE checked: ☐

Data entered: ___ ___ /___ ___ / 20___ ___

Entered by: ______________________________

THANK YOU FOR TAKING PART IN THIS STUDY

SECTION ONE: Violence and abuse

We would like to know if you have experienced any of the following BEHAVIOURS from any partner/ex-partner **since the last questionnaire**. For each statement listed below, please indicate how often you have experienced this since the last questionnaire. We want to know about all of these experiences, even those you may not have considered very serious.

Emotional behaviour

How often have you experienced any of the following emotional abuse from any partner/ex-partner **since the last questionnaire**?

	Never	Sometimes	Often
Isolated from friends or family			
Told what to do/not do, where to go/not go, who to see/not see			
Made you feel you had to ask permission to do certain things such as going out, seeing friends, etc (above and beyond being polite)			
Made you feel afraid by things they did/said			
Prevented you from leaving the home			
Controlled the family/household money			
Threats to hurt you			
Extreme jealousy or possessiveness			
Told you what to wear or not to wear or how to look			
Humiliated/embarrassed you in front of others			

Something else relating to emotional behaviour (please describe)

SECTION ONE: Violence and abuse

Emotional behaviour

As well as knowing about your experiences of certain behaviours we want to know if you have engaged in any of these behaviours TOWARDS any partner/ex-partner **since completing the last questionnaire**

How often have you done the following to any partner/ most ex-partner **since completing the last questionnaire**?

	Never	Sometimes	Often
Isolated from friends or family			
Told partner what to do/not do, where to go/not go, who to see/not see			
Made partner feel they had to ask permission to do certain things such as going out, seeing friends, etc (above and beyond being polite)			
Made them feel afraid by things you did/said			
Prevented partner/ex-partner from leaving the home			
Controlled the family/household money			
Threats to hurt partner/ex-partner			
Extreme jealousy or possessiveness			
Told partner/ex-partner what to wear or not to wear or how to look			
Humiliated/embarrassed partner/ex-partner in front of others			

Something else relating to emotional behaviour (please describe)

SECTION ONE: Violence and abuse

Physical behaviour

How often have you experienced the following physical abuse from any partner/ex-partner **since completing the last questionnaire**?

	Never	Sometimes	Often
Slapped/pushed/shoved			
Kicked/punched			
Beaten up			
Burned			
Bitten			
Restrained/held down/tied up			
Put their hands on your throat or neck			
Hit with object or weapon			
Threatened with object/weapon			
Threatened to kill			
Prevented you getting help for injuries			
Stalked/followed/harassed you			
Locked in house or room			

Something else relating to physical behaviour (please describe)

SECTION ONE: Violence and abuse

Physical behaviour

As well as knowing about your experiences of certain behaviours we want to know if you have engaged in any of these behaviours TOWARDS any partner/ex-partner **since completing the last questionnaire**.

How often have you done the following to any partner/ most recent ex-partner **since completing the last questionnaire**?

	Never	Sometimes	often
Slapped/pushed/shoved			
Kicked/punched			
Beaten up			
Burned			
Bitten			
Restrained/held them down/tied them up			
Put your hands on their throat or neck (trying to choke or strangle or suffocate)			
Hit with object or weapon			
Threatened with object/weapon			
Threatened to kill			
Prevented them from getting help for injuries			
Stalked/followed/harassed them			
Locked them in house or room			

Something else relating to physical behaviour (please describe)

SECTION ONE: Violence and abuse

Sexual behaviour

How often have you experienced the following sexual abuse from a partner/ex-partner **since completing the last questionnaire**?

	Never	Sometimes	Often
Touched in a way which caused fear/alarm/distress			
Forced into doing something sexual you didn't want to			
Hurt during sex			
Had boundaries or safe words disrespected			
Made to have sex when you didn't want to or didn't stop when you wanted to			
Sexually assaulted or abused in any way			
Threats to sexually assault/abuse you			

Something else relating to sexual behaviour (please describe)

SECTION ONE: Violence and abuse

Sexual behaviour

As well as knowing about your experiences of certain behaviours we want to know if you have engaged in any of these behaviours TOWARDS any partner/ex-partner **since completing the last questionnaire**

How often have you done the following to any partner/ ex-partner **since completing the last questionnaire**?

	Never	Sometimes	Often
Touched in a way which caused fear/alarm/distress			
Forced into doing something sexual they didn't want to			
Hurt them during sex			
Disrespected boundaries or safe words			
Made them have sex when they didn't want to or didn't stop when they wanted you to			
Sexually assaulted or abused them in any way			
Threatened to sexually assault/abuse			

Something else relating to sexual behaviour (please describe)

SECTION ONE: Violence and abuse

Impact of abusive behaviour you have experienced on you

Please tick all of the ways any of the above behaviour affected you.

1☐ Injuries such as bruises/scratches/minor cuts

2☐ Injuries needing help from doctor/hospital

3☐ Didn’t have an impact

4☐ Lost respect for your partner

5☐ Made you want to leave partner

6☐ Depression/sleeping problems

7☐ Stopped trusting partner

8☐ Felt unable to cope

9☐ Felt worthless or lost confidence

10☐ Felt sadness

11☐ Felt anxious/panic/lost concentration

12☐ Felt isolated/stopped going out

13☐ Felt angry/shocked

14☐ Self-harmed/felt suicidal

15☐ Worried partner might leave

16☐ Defended self/children/pets

17☐ Feared for life

18☐ Felt had to watch what you say/do

19☐ Not applicable

20☐ Something else relating to the impact of the abusive behaviour – please say what

SECTION ONE: Violence and abuse

If you answered questions above about behaving in an abusive way towards your partner/ex-partner what impact do you think your behaviour had on your partner/ex-partner?

1☐ Injuries such as bruises/scratches/minor cuts

2☐ Injuries needing help from doctor/hospital

3☐ Didn't have an impact

4☐ They lost respect for you

5☐ Made them want to leave you

6☐ Depression/Sleeping problems

7☐ They stopped trusting you

8☐ They felt unable to cope

9☐ Felt worthless or lost confidence

10☐ Felt sadness

11☐ Felt anxious/panic/lost concentration

12☐ Felt isolated/stopped going out

13☐ Felt angry/shocked

14☐ Self-harmed/felt suicidal

15☐ Feared for their life

16☐ They had to be careful of what they said/did

17☐ Not applicable

18☐ Something else relating to the impact of the abusive behaviour – please say what

Why do you think you did any of these things:
Please tick all that apply.

1 ☐ To stop them from doing something

2 ☐ Made you feel in control

3 ☐ Because they were laughing at you

4 ☐ Because they betrayed/rejected you

5 ☐ To make them do something you wanted them to do

6 ☐ Because you didn't trust them

7 ☐ Because of your alcohol/drug use

8 ☐ To stop them from leaving you

9 ☐ Didn't feel good enough/felt insecure

10 ☐ Because you were jealous/possessive

11 ☐ Not applicable

12 ☐ Some other reason – please say what:

Since completing the last questionnaire, how often have the police been called to your house because of violence/abuse any partner/ex-partner was using?

1☐ Not at all   2☐ Once   3☐ 2-5 times   4☐ 6-10 times

5☐ More than 10 times

SECTION TWO: Your health and well-being

Sub-section A

Do you have any current or past mental health problems? (Please include any diagnosis you have been given)

0☐ No 1☐ Yes

If your answer is yes, please describe

Have you had any treatment or support for these current or past mental health problems?

0☐ No 1☐ Yes

If your answer is yes, please describe

SECTION TWO: Your health and well-being

Sub-section B

This survey asks for your views about your health. This information will help keep track of how you feel and how well you are able to do your usual activities. Answer each question by selecting the answer as indicated. If you are unsure how to answer a question, please give the best answer you can.

	**Excellent**	**Very good**	**Good**	**Fair**	**Poor**
1. In general, would you say your health is	1☐	2☐	3☐	4☐	5☐

2. The following questions are about activities you might do during a typical day. Does your health now limit you in these activities? If so, how much?

	**Yes, limited a lot**	**Yes, limited a little**	**No, not limited at all**
a. Moderate activities such as moving a table, pushing a vacuum cleaner, bowling or playing golf	1☐	2☐	3☐
b. Climbing several flights of stairs	1☐	2☐	3☐

3. During the **past 4 weeks**, how much of the time have you had any of the following problems with your work or other regular daily activities as a result of your physical health?

	**All of the time**	**Most of the time**	**Some of the time**	**A little of the time**	**None of the time**
a. Accomplished less than you would like	1☐	2☐	3☐	4☐	5☐
b. Were limited in the kind of work or other activities	1☐	2☐	3☐	4☐	5☐

4. During the **past 4 weeks**, how much of the time have you had any of the following problems with your work or other regular daily activities as a result of any emotional problems (such as feeling depressed or anxious)?

	**All of the time**	**Most of the time**	**Some of the time**	**A little of the time**	**None of the time**
a. Accomplished less than you would like	1☐	2☐	3☐	4☐	5☐
b. Did work or activities less carefully than usual	1☐	2☐	3☐	4☐	5☐

5. During the past **4 weeks**, how much did pain interfere with your normal work (including both work outside the home and housework)?

**Not at all**	**A little bit**	**Moderately**	**Quite a bit**	**Extremely**
1☐	2☐	3☐	4☐	5☐

6. These questions are about how you feel and how things have been with you during the **past 4 weeks**. For each question, please give the one answer that comes closest to the way you have been feeling. How much of the time **during the past 4 weeks**…

	**All of the time**	**Most of the time**	**Some of the time**	**A little of the time**	**None of the time**
a. Have you felt calm and peaceful?	1☐	2☐	3☐	4☐	5☐
b. Did you have a lot of energy?	1☐	2☐	3☐	4☐	5☐
c. Have you felt downhearted and depressed?	1☐	2☐	3☐	4☐	5☐

7. During the **past 4 weeks**, how much of the time has your physical health or emotional problems interfered with your social activities (like visiting friends, relatives etc.)?

All of the time	Most of the time	Some of the time	A little of the time	None of the time
1☐	2☐	3☐	4☐	5☐

SECTION TWO: Your health and well-being

Sub-section C

Under each heading, please tick the ONE box that best describes your health TODAY.

MOBILITY

1 ☐ I have no problems in walking about

2 ☐ I have slight problems in walking about

3 ☐ I have moderate problems in walking about

4 ☐ I have severe problems in walking about

5 ☐ I am unable to walk about

SELF-CARE

1 ☐ I have no problems washing or dressing myself

2 ☐ I have slight problems washing or dressing myself

3 ☐ I have moderate problems washing or dressing myself

4 ☐ I have severe problems washing or dressing myself

5 ☐ I am unable to wash or dress myself

USUAL ACTIVITIES (*e.g. work, study, housework, family or leisure activities)*

1 ☐ I have no problems doing my usual activities

2 ☐ I have slight problems doing my usual activities

3 ☐ I have moderate problems doing my usual activities

4 ☐ I have severe problems doing my usual activities

5 ☐ I am unable to do my usual activities

PAIN/DISCOMFORT

1 ☐ I have no pain or discomfort

2 ☐ I have slight pain or discomfort

3 ☐ I have moderate pain or discomfort

4 ☐ I have severe pain or discomfort

5 ☐ I have extreme pain or discomfort

ANXIETY/DEPRESSION

1☐ I am not anxious or depressed

2☐ I am slightly anxious or depressed

3☐ I am moderately anxious or depressed

4☐ I am severely anxious or depressed

5☐ I am extremely anxious or depressed

- We would like to know how good or bad your health is TODAY.
- This scale is numbered 0 to 100.
- 100 means the <u>best</u> health you can imagine.
- 0 means the <u>worst</u> health you can imagine.
- Mark an X on the scale to indicate how your health is TODAY.
- Now, please write the number you marked on the scale in the box below.

Your health today = ☐

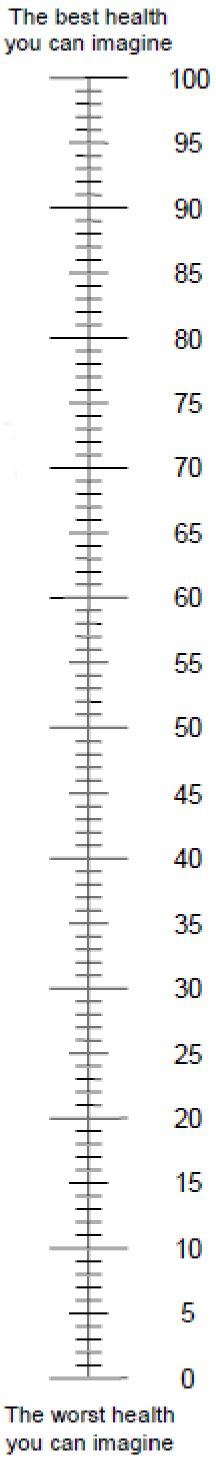

<u>SECTION TWO: Your health and well-being</u>

<u>Sub-section D</u>

<u>Over the past 2 weeks</u>, how often have you been bothered by any of the following problems? Please tick the response that best describes you.

	Not at all	Several days	More than half the days	Nearly every day
1. Little interest or pleasure in doing things	0☐	1☐	2☐	3☐
2. Feeling down, depressed, or hopeless	0☐	1☐	2☐	3☐
3. Trouble falling or staying asleep, or sleeping too much	0☐	1☐	2☐	3☐
4. Feeling tired or having little energy	0☐	1☐	2☐	3☐
5. Poor appetite or overeating	0☐	1☐	2☐	3☐
6. Feeling bad about yourself - or that you're a failure or have let yourself or your family down	0☐	1☐	2☐	3☐
7. Trouble concentrating on things, such as reading the newspaper or watching television	0☐	1☐	2☐	3☐
8. Moving or speaking so slowly that other people could have noticed? Or, the opposite - being so fidgety or restless that you have been moving around a lot more than usual	0☐	1☐	2☐	3☐
9. Thoughts that you would be better off dead, or of hurting yourself in some way	0☐	1☐	2☐	3☐

10. If you have ticked any problems, how difficult have those problems made it for you to do your work, take care of things at home, or get along with other people?

0☐ Not difficult at all 1☐ Somewhat difficult 2☐ Very difficult

3☐ Extremely difficult

SECTION TWO: Your health and well-being

Sub-section E

Over the last 2 weeks, how often have you been bothered by the following problems? Please tick the response that best describes you.

	Not at all	Several days	Over half the days	Nearly every day
1. Feeling nervous, anxious, or on edge	0☐	1☐	2☐	3☐
2. Not being able to stop or control worrying	0☐	1☐	2☐	3☐
3. Worrying too much about different things	0☐	1☐	2☐	3☐
4. Trouble relaxing	0☐	1☐	2☐	3☐
5. Being so restless that it is hard to sit still	0☐	1☐	2☐	3☐
6. Becoming easily annoyed or irritable	0☐	1☐	2☐	3☐
7. Feeling afraid as if something awful might happen	0☐	1☐	2☐	3☐

If you ticked off any problems, how difficult have these made it for you to do your work, take care of things at home, or get along with other people?

0☐ Not difficult at all 1☐ Somewhat difficult 2☐ Very difficult

3☐ Extremely difficult

SECTION TWO: Your health and well-being

Sub-section F

Below is a list of problems that people sometimes have in response to a very stressful experience. Please read each problem carefully and then tick one of the boxes to the right to indicate how much you have been bothered by that problem in the past month.

In the past month, how much were you bothered by:

	Not at all	A little bit	Moderately	Quite a bit	Extremely
1. Repeated, disturbing, and unwanted memories of the stressful experience?	0☐	1☐	2☐	3☐	4☐
2. Repeated, disturbing dreams of the stressful experience?	0☐	1☐	2☐	3☐	4☐
3. Suddenly feeling or acting as if the stressful experience were actually happening again (as if you were actually back there reliving it)?	0☐	1☐	2☐	3☐	4☐
4. Feeling very upset when something reminded you of the stressful experience?	0☐	1☐	2☐	3☐	4☐
5. Having strong physical reactions when something reminded you of the stressful experience (for example, heart pounding, trouble breathing, sweating)?	0☐	1☐	2☐	3☐	4☐
6. Avoiding memories, thoughts, or feelings related to the stressful experience?	0☐	1☐	2☐	3☐	4☐
7. Avoiding external reminders of the stressful experience (for example,	0☐	1☐	2☐	3☐	4☐

people, places, conversations, activities, objects, or situations)?					
	**Not at all**	**A little bit**	**Moderately**	**Quite a bit**	**Extremely**
8. Trouble remembering important parts of the stressful experience?	0☐	1☐	2☐	3☐	4☐
9. Having strong negative beliefs about yourself, other people, or the world (for example, having thoughts such as: I am bad, there is something seriously wrong with me, no one can be trusted, the world is completely dangerous)?	0☐	1☐	2☐	3☐	4☐
10. Blaming yourself or someone else for the stressful experience or what happened after it?	0☐	1☐	2☐	3☐	4☐
11. Having strong negative feelings such as fear, horror, anger, guilt, or shame?	0☐	1☐	2☐	3☐	4☐
12. Loss of interest in activities that you used to enjoy?	0☐	1☐	2☐	3☐	4☐
13. Feeling distant or cut off from other people?	0☐	1☐	2☐	3☐	4☐
14. Trouble experiencing positive feelings (for example, being unable to feel happiness or have loving feelings for people close to you)?	0☐	1☐	2☐	3☐	4☐
15. Irritable behaviour, angry outbursts, or acting aggressively?	0☐	1☐	2☐	3☐	4☐
16. Taking too many risks or doing things that could cause you harm?	0☐	1☐	2☐	3☐	4☐
17. Being "superalert" or watchful or on guard?	0☐	1☐	2☐	3☐	4☐
18. Feeling jumpy or easily startled?	0☐	1☐	2☐	3☐	4☐
19. Having difficulty concentrating?	0☐	1☐	2☐	3☐	4☐
20. Trouble falling or staying asleep?	0☐	1☐	2☐	3☐	4☐

Sub-section G

ABOUT YOUR OVERALL QUALITY OF LIFE

Please indicate which statements best describe your overall quality of life at the moment by placing a tick (✓) in **ONE** box for each of the five groups below.

**1. Feeling settled and secure**		
I am able to feel settled and secure in **all** areas of my life	☐	4
I am able to feel settled and secure in **many** areas of my life	☐	3
I am able to feel settled and secure in **a few** areas of my life	☐	2
I am **unable** to feel settled and secure in **any** areas of my life	☐	1
**2. Love, friendship and support**		
I can have **a lot** of love, friendship and support	☐	4
I can have **quite a lot** of love, friendship and support	☐	3
I can have **a little** love, friendship and support	☐	2
I **cannot** have **any** love, friendship and support	☐	1
**3. Being independent**		
I am able to be **completely** independent	☐	4
I am able to be independent in **many** things	☐	3
I am able to be independent in **a few** things	☐	2
I am **unable** to be at all independent	☐	1
**4. Achievement and progress**		
I can achieve and progress in all aspects of my life	☐	4
I can achieve and progress in **many** aspects of my life	☐	3
I can achieve and progress in **a few** aspects of my life	☐	2
I **cannot** achieve and progress in **any** aspects of my life	☐	1
**5. Enjoyment and pleasure**		
I can have **a lot** of enjoyment and pleasure	☐	4
I can have **quite a lot** of enjoyment and pleasure	☐	3
I can have **a little** enjoyment and pleasure	☐	2
I **cannot** have **any** enjoyment and pleasure	☐	1

Please ensure you have only ticked **ONE** box for each of the five groups.

© 2010 Hareth Al-Janabi and Joanna Coast

SECTION THREE: Your children (if you do not have children please go to section Four)

Which of the following currently applies to your children?
Tick all those which apply.

1☐ The courts or state child protection have told my partner/ex-partner he/she can't live with our children

2☐ The courts or state child protection have stopped me from living with my children

3☐ The courts or state child protection have stopped him/her having contact/access

4☐ The courts or state child protection have stopped me having contact/access

5☐ I have applied to the court for contact with our children

6☐ My partner/ex-partner has applied to the court for contact with our children

7☐ My children have been removed and are being looked after by foster carers

8☐ I don't think our children were affected by the abuse

9☐ My partner/ex-partner doesn't think our children were affected by the abuse

10☐ One or more of my children is angry or upset with me

11☐ One or more of my children is angry/upset with my partner/ex-partner because of what's happened

12☐ One or more of my children is currently registered with the state child protection as in need of protection because of the violence/abuse in our relationship

13☐ Not applicable

14☐ Something else in relation to your children:

60

<u>Does your child / any of your children currently have any disabilities or special educational needs?</u>

☐ No ☐ Yes ☐ N/A

<u>If yes, please briefly describe your child's/children's disabilities or special educational needs</u>

SECTION THREE: Your children

Your children's health and wellbeing

If you **do not** have any children aged between 8-18 then **please go to Section Four.**

If you have any **children aged between 8-18,** please fill out the following section thinking about the **child whose birthday is first in the calendar year**.

How is your child? How does she/he feel? This is what we would like to know from you.

Please answer the following questions to the best of your knowledge, ensuring that the answers you give reflect the perspective of the selected child. Please try to remember your child's experiences over the last week...

What is your relationship to the child?

1☐ Mother
2☐ Father
3☐ Stepmother/father's partner
4☐ Stepfather/mother's partner
3☐ Other

Please state relationship

☐

How old is your child (the selected child)?

☐☐ years

Is your child (the selected child) female or male?

1☐ Female
2☐ Male
3☐ If neither of the above please specify you child's preference for gender

SECTION THREE: Your children

Physical Activities and Health

1. In general, how would your child rate her/his health?

1☐ Excellent

2☐ Very good

3☐ Good

4☐ Fair

5☐ Poor

Thinking about the last week...

2. Has your child felt fit and well?	**Not at all** 1☐	**Slightly** 2☐	**Moderately** 3☐	**Very** 4☐	**Extremely** 5☐
3. Has your child been physically active (e.g. running, climbing, biking)?	**Not at all** 1☐	**Slightly** 2☐	**Moderately** 3☐	**Very** 4☐	**Extremely** 5☐
4. Has your child been able to run well?	**Not at all** 1☐	**Slightly** 2☐	**Moderately** 3☐	**Very** 4☐	**Extremely** 5☐
5. Has your child felt full of energy?	**Never** 1☐	**Seldom** 2☐	**Quite often** 3☐	**Very often** 4☐	**Always** 5☐

General Mood and Your Child's Feelings
Thinking about the last week…

1. Has your child felt that life was enjoyable?	**Not at all**<br>1☐	**Slightly**<br>2☐	**Moderately**<br>3☐	**Very**<br>4☐	**Extremely**<br>5☐
2. Has your child been in a good mood?	**Never**<br>1☐	**Seldom**<br>2☐	**Quite often**<br>3☐	**Very often**<br>4☐	**Always**<br>5☐
3. Has your child had fun?	**Never**<br>1☐	**Seldom**<br>2☐	**Quite often**<br>3☐	**Very often**<br>4☐	**Always**<br>5☐
4. Has your child felt sad?	**Never**<br>1☐	**Seldom**<br>2☐	**Quite often**<br>3☐	**Very often**<br>4☐	**Always**<br>5☐
5. Has your child felt so bad that he/she didn't want to do anything?	**Never**<br>1☐	**Seldom**<br>2☐	**Quite often**<br>3☐	**Very often**<br>4☐	**Always**<br>5☐
6. Has your child felt lonely?	**Never**<br>1☐	**Seldom**<br>2☐	**Quite often**<br>3☐	**Very often**<br>4☐	**Always**<br>5☐
7. Has your child been happy with the way he/she is?	**Never**<br>1☐	**Seldom**<br>2☐	**Quite often**<br>3☐	**Very often**<br>4☐	**Always**<br>5☐

Family and Your Child's Free Time

Thinking about the last week...

1. Has your child had enough time for him/herself?	**Never**<br>1☐	**Seldom**<br>2☐	**Quite often**<br>3☐	**Very often**<br>4☐	**Always**<br>5☐
2. Has your child been able to do the things that he/she wants to do in his/her free time?	**Never**<br>1☐	**Seldom**<br>2☐	**Quite often**<br>3☐	**Very often**<br>4☐	**Always**<br>5☐
3. Has your child felt that his/her<br>parent(s) had enough time for him/her?	**Never**<br>1☐	**Seldom**<br>2☐	**Quite often**<br>3☐	**Very often**<br>4☐	**Always**<br>5☐
4. Has your child felt that his/her<br>parent(s) treated him/her fairly?	**Never**<br>1☐	**Seldom**<br>2☐	**Quite often**<br>3☐	**Very often**<br>4☐	**Always**<br>5☐
5. Has your child been able to talk to his/her parent(s) when he/she wanted to?	**Never**<br>1☐	**Seldom**<br>2☐	**Quite often**<br>3☐	**Very often**<br>4☐	**Always**<br>5☐
6. Has your child had enough money to do the same things as his/her friends?	**Never**<br>1☐	**Seldom**<br>2☐	**Quite often**<br>3☐	**Very often**<br>4☐	**Always**<br>5☐
7. Has your child felt that he/she had enough money for his/her expenses?	**Never**<br>1☐	**Seldom**<br>2☐	**Quite often**<br>3☐	**Very often**<br>4☐	**Always**<br>5☐

Friends

Thinking about the last week...

1. Has your child spent time with his/her friends?	**Never** 1☐	**Seldom** 2☐	**Quite often** 3☐	**Very often** 4☐	**Always** 5☐
2. Has your child had fun with his/her friends?	**Never** 1☐	**Seldom** 2☐	**Quite often** 3☐	**Very often** 4☐	**Always** 5☐
3. Have your child and his/her friends helped each other?	**Never** 1☐	**Seldom** 2☐	**Quite often** 3☐	**Very often** 4☐	**Always** 5☐
4. Has your child been able to rely on his/her friends?	**Never** 1☐	**Seldom** 2☐	**Quite often** 3☐	**Very often** 4☐	**Always** 5☐

School and Learning

Thinking about the last week...

1. Has your child been happy at school?	**Not at all** 1☐	**Slightly** 2☐	**Moderately** 3☐	**Very** 4☐	**Extremely** 5☐
2. Has your child got on well at school?	**Not at all** 1☐	**Slightly** 2☐	**Moderately** 3☐	**Very** 4☐	**Extremely** 5☐
3. Has your child been able to pay attention?	**Never** 1☐	**Seldom** 2☐	**Quite often** 3☐	**Very often** 4☐	**Always** 5☐

4. Has your child got along well with his/her teachers?	**Never**	**Seldom**	**Quite often**	**Very often**	**Always**
	1☐	2☐	3☐	4☐	5☐

Anything else about your child's health or wellbeing

SECTION FOUR: Your partner/ex-partner and your relationship

At the moment, how often are you fearful of your partner/ex-partner?

0☐ Never 1☐ Not often 2☐ Sometimes 3☐ Often 4☐ Always/mostly

What is your relationship status with your partner/ex-partner NOW?

1☐ Together and living together

2☐ Together but living apart

3☐ In the process of splitting up

4☐ The relationship has ended and we are living apart with no contact

5☐ The relationship has ended and we are living apart and still have contact

6☐ I am not sure

7☐ Something else – please say:

What are your hopes for your relationship with them in the future?

1☐ That we will be together and living together

2☐ That this relationship will end

3☐ I am not sure

4☐ I am in another relationship already

5☐ Something else – please say what:

SECTION FIVE: Your use of health services

This section asks for information about YOUR recent use of health and social care services since the last questionnaire

	**No**<br>Please go to question **2**	**Yes**<br>Please answer question **1a**
1. **Have you been admitted to hospital overnight for any reason since completing the last questionnaire?**	1☐	2☐

1a. If YES, please tell us the reason you were admitted and how many nights you were in hospital in the boxes below. If you had more than one admission please complete details for each admission in a separate box

Admission 1

Reason for admission — No. of nights in hospital ☐☐

....

Admission 2

Reason for admission — No. of nights in hospital ☐☐

....

Admission 3

Reason for admission — No. of nights in hospital ☐☐

....

Admission 4

Reason for admission — No. of nights in hospital ☐☐

……………………………………………………………………………………

2. Have you used any of the following outpatient services at any hospital? An outpatient appointment is when you attend the hospital for treatment without being admitted overnight. If you answer YES to any of these, please tell us the number of appointments you have had since you completed the last questionnaire

**Name of service**	**I have used this service**<br><br>**No**	 <br>**Yes**	**No. of appointments/ attendances**
a. A&E attendance (no ambulance used)	☐	☐	
b. A&E attendance (ambulance used)	☐	☐	
c. Other outpatient appointment (e.g. Ear Nose and Throat, Neurology, Gynaecology, Physiotherapy, Cardiology) please specify<br>……………………………………………………………………………………<br>……………………………………………………………………………………	☐	☐	

3. Have you used any of the following health services? If you answer YES to any of these, please tell us the number of appointments you have had since you completed the last questionnaire

**Name of service**	**I have used this service**<br>**No**	**Yes**	**No. of appointments**
a. General practitioner (GP) - face to face meeting at the surgery	☐	☐	
b. General practitioner (GP) – face to face meeting at home	☐	☐	
c. General practitioner (GP) – telephone consultation/conversation	☐	☐	
d. Practice nurse – face to face at the surgery	☐	☐	
e. Practice nurse – telephone consultation/conversation	☐	☐	
f. District nurse	☐	☐	
g. Other nurse (e.g. health visitor, midwife)<br>please specify:<br>..	☐	☐	
h. NHS direct	☐	☐	
i. NHS walk-in centre	☐	☐	
j. Other community based doctor (please specify)<br>..	☐	☐	
k. Occupational or physiotherapist	☐	☐	

4. Have you used any of the following mental health services? If you answer YES to any of these, please tell us the number of appointments you have had since you completed the last questionnaire

**Name of service**	**I have used this service**<br><br>**No**	 <br><br>**Yes**	**No. of appointments**
a. Community mental health nurse/care coordinator	☐	☐	
b. Psychiatrist (NOT seen as inpatient)<br>Psychiatrists diagnose and prescribe medication for mental health conditions.	☐	☐	
c. Psychologist (NOT seen as inpatient)<br>Psychologists provide therapy and counselling. They do not prescribe medication or give diagnoses.	☐	☐	
d. Crisis resolution/Home treatment team	☐	☐	
e. Other mental health worker (e.g. Primary care mental health worker)<br>Please specify:<br>……………………………………………………………………………<br>……………………………………………………………………………	☐	☐	

**5. Have you used any of the following therapeutic services? If you answer YES to any of these, please tell us the number of appointments you have had since you completed the last questionnaire and an approximate cost per appointment, if you have had to pay.**				
**Name of service**	**I have used this service**		**No. of appointments/ sessions?**	**Total cost per appointment/ sessions? (if no cost to self, put 0)**
	**No**	**Yes**		
a. Counsellor – based in the GP practice	☐	☐		£
b. Counsellor – based with a voluntary sector organisation	☐	☐		£
c. Counsellor – based elsewhere (please specify)	☐	☐		£
d. Family therapist	☐	☐		£
e. Other talking therapy (please specify)				£
f. Complementary or alternative therapy (e.g. acupuncture, homeopathy) Please specify:	☐	☐		£
g. Other therapeutic physical or recreational activities (e.g. mindfulness) Please specify:	☐	☐		£

6. Have you used any self-help material (e.g. self-help books, CDs, computer programmes.			
**Please specify**	**Yes**	**No**	**Estimate cost (if no cost to self, put 0)**
……………………………………………………………………………………………………<br>……………………………………………………………………………………………………<br>……………………………………………………………………………………………………<br>……………………………………………………………………………………………………	☐	☐	£

7. Have you used any of the following social care or other community services? If you answer YES to any of these, please tell us the number of appointments you have had since you completed the last questionnaire			
**Name of service**	**I have used this service**<br>**No**	 **Yes**	**No. of appointments**
a. Social worker/care manager	☐	☐	
b. Community support worker	☐	☐	
c. Housing worker (local authority)	☐	☐	
d. Drug & alcohol team	☐	☐	
e. Home help/home care worker	☐	☐	
f. Other community service (e.g. Sure Start) Please specify:<br>………………………………………………………………………	☐	☐	

8. Have you used any of the following voluntary sector services? If you answer YES to any of these, please tell us the number of appointments you have had since you completed the last questionnaire		
**Name of service**	**I have used this service**<br>**No**	 **Yes**
a. Next Link, RSVP or Women's Aid (or any other domestic violence or abuse support service)	☐	☐
b. Freedom programme	☐	☐
c. Other group (e.g. anger management, Building Better Relationships (BBR), mindfulness etc) (please specify)<br>…………………………………………………………………………	☐	☐
d. Day care/drop in/social club (please specify)<br>…………………………………………………………………………	☐	☐
e. Rape crisis or other sexual assault service	☐	☐
f. Helplines (e.g. national domestic violence helpline, Samaritans, RESPECT's men's line) please specify:<br>…………………………………………………………………………	☐	☐
g. Citizen's Advice Bureau	☐	☐
h. Debt advice charity/debt advisor	☐	☐
i. Other voluntary sector organisation (e.g. housing support worker, employment support worker or any other support worker)<br>…………………………………………………………………………	☐	☐

**9. Have you lived in any temporary accommodation since you completed the last questionnaire (<u>If no please go to question 10</u>).**			
**Accommodation**	**I have used this accommodation**<br>**No**	**Yes**	**Length of time in weeks (to the nearest week)**
a. Hostel	☐	☐	
b. Refuge	☐	☐	
c. Sheltered accommodation	☐	☐	
d. Supported living	☐	☐	
e. Bed and breakfast	☐	☐	
f. Sleeping rough	☐	☐	
g. Staying with friends/family	☐	☐	
h. Other<br>……………………………………………………………………<br>……………………………………………………………………<br>……………………………………………………………………<br>……………………………………………………………………	☐	☐	

10. Since you completed the last questionnaire, have you and your child/ren been placed in a family foster placement (e.g. you and your child/ren were placed together with a foster carer)?	
**No** ☐ **Yes** ☐	**How long for in weeks?**

11.Since you completed the last questionnaire, has your child/ren been placed by the local authority in any kind of foster placement?	
**No** ☐ **Yes** ☐	**How long for in weeks?**

12. Since you completed the last questionnaire have you had to move house?

1☐ No → Please go to question **13**

2☐ Yes

Have had to….?	No	Yes	Estimate cost (if no cost to self, put zero)
a. Hire a van/car to move house?	☐	☐	£
b. Pay additional rent/deposit or mortgage payments?	☐	☐	£
c. Buy any new furniture or household items? ……………………………………………………………	☐	☐	£
d. Have any other relocation costs (e.g. new school uniforms for children, childcare whilst moving, needing to hire someone to help you move, having to pay additional money in transport costs from new home, redirection of mail)	☐	☐	£

13. Since you completed the last questionnaire, have you had to make any changes to your house?		
1☐ No → Please go to question **14a**<br>2☐ Yes		
**Have you had to…?**	**No Yes**	**Estimate cost (if no cost to self, put zero)**
a. Change locks?	☐ ☐	£
b. Replace, refurbish or dispose of damaged furniture?	☐ ☐	£
c. Replace windows or doors?	☐ ☐	£
d. Pay for any other household damage? Please specify<br>…………………………………………………………<br>…………………………………………………………	☐ ☐	£

14a. Employment
**Since you completed the last questionnaire have you had any time off work?**
1☐ No<br>2☐ Yes → Please answer question **14b**
**14b. If YES, was this paid or unpaid time off?**
1☐ Paid → Number of days off work ☐<br>2☐ Unpaid → Number of days off work ☐

**15a. Legal**<br><br>**Since completing the last questionnaire, have you had to instruct a solicitor in relation to your current (or previous) relationship or your children? (e.g. contact order, residence order, non-molestation order)?**
1☐ No<br>2☐ Yes ⟶ Please answer question 15b
**15b. If YES, did you receive legal aid?**
1☐ No<br>2☐ Yes

**16. Childcare costs**<br><br>**Since completing the last questionnaire, have you had any additional child costs (which have not been specified so far)?**		
**No Yes**	**Please specify (e.g. one week's extra nursery session due to house move, one day per week extra after school club)**	**Estimate cost (if no cost to self, put zero)**
☐ ☐	……………………………………………………………………………………<br>……………………………………………………………………………………<br>……………………………………………………………………………………<br>……………………………………………………………………………………<br>……………………………………………………………………………………	£

17. Children

This question asks about your children's use of health and support services. <u>If you do not have children then please go to question 18</u>

Since completing the last questionnaire, have any of your child/ren used any health or support services in relation to:			
	**No**	**Yes**	**Total number of appointments for all children**
a. Mental health problem	☐	☐	
b. Emotional/ behaviour difficulties	☐	☐	
c. Unexplained or chronic pain	☐	☐	
d. Sleep problems	☐	☐	

**18. Other**<br>**Since completing the last questionnaire, have you had any other additional personal or social costs (which have not been specified above)?**	
**Please specify**	**Estimate cost (if no cost to self, put zero)**
……………………………………………………<br>……………………………………………………<br>……………………………………………………<br>……………………………………………………	£

Final thoughts

Is there anything else you want to tell us about your circumstances or stressful events you might have experienced over the last year?	

Thank you for completing this questionnaire. Please give it/send it back to the researcher who gave it to you.

THANK YOU FOR COMPLETING THIS QUESTIONNAIRE!f you have any comments please write them here.
